# Atovaquone-induced activation of the PERK/eIF2α signaling axis mitigates metabolic radiosensitisation

**DOI:** 10.1186/s12964-025-02160-9

**Published:** 2025-04-02

**Authors:** Jie Feng, Varun Pathak, Niall M. Byrne, Sarah Chambers, Tongchuan Wang, Rayhanul Islam, Reinhold J. Medina, Jonathan A. Coulter

**Affiliations:** 1 School of Pharmacy, Queen’s University Belfast, BT9 7BL Belfast, Northern Ireland, UK; 2 Welcome-Wolfson Institute for Experimental medicine, Queen’s University Belfast, Belfast, Northern Ireland, UK; 3 Department of Eye and Vision Sciences, Institute for Life Course and Medical Sciences, University of Liverpool, Liverpool, UK

**Keywords:** Hypoxia, Autophagy, ER stress, Radiosensitisation

## Abstract

**Background:**

Hypoxia, a key feature of most solid tumours, including head and neck cancer, reduces radiotherapy efficacy by promoting radiation resistance through micro-environmental and genomic alterations. Addressing these resistance mechanisms is crucial, as radiotherapy remains central to managing locally advanced disease. Atovaquone, a mitochondrial electron transport chain complex III inhibitor, is reported to reduce tumour hypoxia in preclinical models, however, this response does not consistently enhance radiation sensitivity. This work examines the potential of atovaquone to modify the hypoxic response in models of head and neck squamous cell carcinoma (HNSCC), uncovering an adaptive resistance mechanism driven by integrated stress response (ISR) signaling that limits the radiosensitising potential of this approach.

**Methods:**

The bioenergetic response of HNSCC cells to atovaquone was assessed using the Seahorse XFe96 Analyzer with the XF Cell Mito Stress Test. Radiation dose modifying effects of atovaquone were tested by clonogenic survival assays, while ROS yields were analysed by flow cytometry. Western blotting and quantitative reverse transcription-PCR were employed to study activation of ISR signaling and the overall influence of atovaquone on the hypoxic response. Finally, the role of the ISR activation in modulating radiosensitivity was investigated using both siRNA and pharmacological inhibition of eIF2α, a central regulator of the ISR.

**Results:**

Herein we report that atovaquone significantly disrupts mitochondrial respiration, triggering phosphorylation of eIF2α, a pivotal regulator of the ISR, and a master regulator of protein synthesis. Notably, atovaquone also increased the autophagic load under hypoxia, while autophagy inhibition significantly enhanced apoptosis, improving radiation sensitivity. Combined eIF2α inhibition and atovaquone promotes cell cycle redistribution and significantly enhances mitochondrial ROS production and compared to atovaquone alone, restoring atovaquone mediated radiosensitisation.

**Conclusions:**

Our data highlight dual counter opposing impacts of atovaquone, serving as a hypoxic radiosensitiser though oxidative phosphorylation (OXPHOS) inhibition, but also in promoting stress induced ISR signaling, conferring resistance to radiation treatment. Importantly, if ISR activation is impeded, the metabolic radiosensitising properties of atovaquone is restored. These data provide a new insight to a molecular response that could help counteract hypoxia-induced radioresistance.

**Supplementary Information:**

The online version contains supplementary material available at 10.1186/s12964-025-02160-9.

## Abstract (plain language summary)

Solid tumours, including head and neck cancer, have poorly oxygenated regions, termed hypoxic, making them less responsive to radiotherapy. Hypoxia not only changes the local tumour environment but also alters the genetic behaviour of the tumour, contributing to treatment resistance. Atovaquone, a drug that inhibits oxygen dependent respiration with the mitochondria, has been shown to sensitise tumour models to radiation by reducing oxygen consumption, a strategy referred to as metabolic radiosensitisation. However, this response is inconsistent between tumour models. In this study, we explored how atovaquone affects the response of head and neck cancer cells to radiation under both normoxic (21% O_2_) and hypoxic (0.5% O_2_) conditions. We found that while atovaquone disrupts mitochondrial energy production, it also activates a stress response pathway called the integrated stress response (ISR). This highly conserved pathway helps protect tumour cells against the metabolism altering properties of atovaquone, ultimately reducing the effectiveness of radiation. Using inhibitors (both drug and genetic) of key proteins in the integrated stress response signaling pathway, tumour cells were rendered more sensitive to radiation, enhancing cell death. These results reveal that while atovaquone has the potential to enhance radiation therapy by reducing hypoxia, it simultaneously triggers protective stress response mechanisms within the tumour cells. By targeting the ISR, we could improve the effectiveness of atovaquone and radiation therapy together. This research offers new strategies to tackle hypoxia-related treatment resistance in cancer.

**Supplementary Information:**

The online version contains supplementary material available at 10.1186/s12964-025-02160-9.

## Introduction

Head and neck squamous cell carcinoma (HNSCC) refers to a group of biologically similar cancers affecting the lips, oral cavity, nasal cavity, pharynx, larynx, and paranasal sinuses [1]. HNSCC is among the sixth most deadly cancers worldwide, with almost 900,000 new diagnosis annually resulting in 450,000 deaths [2]. The incidence of HNSCC continues to rise and by 2030 is expected to increase by 30%. Current clinical intervention includes surgery, radiotherapy, chemotherapy and targeted therapies. For locally advanced HNSCC (LA-HNSCC), radiotherapy remains the standard-of-care [3]. However, effectiveness is hindered by resistance mechanisms, notably hypoxia, leading to unfavourable outcomes [4, 5]. Strategies to counteract hypoxia-mediated radioresistance involve increasing endogenous oxygen levels or targeting tumour hypoxia using hypoxia-activated prodrugs [6]. These approaches, though effective in the pre-clinical setting, failed to deliver patient benefits in the clinic [7]. An alternative approach is to suppress oxygen consumption by inhibiting oxidative phosphorylation (OXPHOS). Pharmacological inhibitors of oxidative metabolism have been proposed to increase tumour oxygenation [8], improving the effectiveness of RT, an effect broadly termed as metabolic radiosensitisation [9]. Atovaquone, an FDA-approved broad-spectrum anti-parasitic drug, inhibits mitochondrial electron transport chain (ETC) function by interfering with ubiquinone-mediated electron transfer at mitochondrial complex III. Recent pre-clinical and clinical studies have reported promising anticancer effects [10, 11]. For example, atovaquone reduced tumour hypoxia by 73.7% in various cancer cell lines, leading to significant tumour growth delay when combined with radiation [12]. Additionally, atovaquone exerts pro-oxidant effects by participating in redox cycling, generating reactive oxygen species [13]. Despite this, a recent study reported limited efficacy of atovaquone alone as a hypoxia radiosensitiser, attributed to significant tumour heterogeneity conferring intrinsic radioresistance [12]. This variability is partly due to mitochondrial stress adaptations, including altered mitochondrial dynamics, metabolism, and apoptosis regulation, promoting therapeutic resistance [14, 15].

ETC inhibition has potential for modulating tumour hypoxia but corresponds with mitochondrial dysfunction triggering the activation of the integrated stress response (ISR) [16]. ISR is regulated by four distinct kinases (PERK, GCN2, PKR and HRI) each activated by specific stressors which converge on phosphorylation of a single serine residue on the α-subunit of eukaryotic translation initiation factor 2 (eIF2) [17]. EIF2α phosphorylation confers general attenuation of translation, while concurrently selectively supporting the synthesis of proteins involved in environmental adaptation, including activating transcriptional factor 4 (ATF4). ATF4, promoted by p-eIF2α is credited as a key protective adaptation to hypoxic stress through autophagy induction and an antioxidant response, contributing to treatment resistance and poor patient outcomes [18–21].

ISR signaling is implicated in protective “yin” and pro-apoptotic “yang” activities across various malignancies [22]. A recent study demonstrated atovaquone induced pro-apoptotic signaling via ATF4 upregulation in an eIF2α dependent manner [23]. However, the underpinning mechanisms by which eIF2α contributes to therapeutic resistance is not well understood. This work demonstrates, for the first time, that atovaquone induces ISR signaling, triggering a cascade of radioprotective responses that ultimately contribute to hypoxic radioresistance. Importantly, inhibition of the eIF2α/ISR axis can restore hypoxic radiosensitivity achieved through OXPHOS inhibition. While focusing exclusively on atovaquone, these data are likely to have broader implication on the wider strategy of metabolic radiosensitisation, while highlighting combined eIF2α/ISR inhibition as a valid target.

## Materials and methods

### Chemical regents and antibodies

Atovaquone, ISRIB (SML0843) were purchased from Sigma-Aldrich (USA). Primary rabbit monoclonal antibodies against phospho-eIF2α (#9721S), phosphor-GCN2 (Thr899) (#94688T), total eIF2α (#9722S), LC3B (#2535S), PERK (#5683S), cyclin D1 (#2978S), α-tubulin (#2144S) were purchased from Cell Signalling Technology. Rabbit monoclonal antibody against HIF-1α (#ab179483), rabbit polyclonal antibody against phospho-PERK(#T981), and goat anti-rabbit IgG H&L (HRP) (ab205718) were obtained from Abcam. Rabbit polyclonal antibody against HRI (#A05465) was purchased from Boster Biological Technology. The Seahorse XF Cell Mito Stress Test Kit was from Agilent Technologies.

### Cell culture

The HPV (-) HNSCC cell line CAL27 was obtained from the American Type Culture Collection (ATCC). FaDu and CAL33 cells were obtained from DSMZ (Braunschweig, Germany). CAL27 cells were grown in Dulbecco’s modified Eagle’s medium (DMEM, Sigma-Aldrich) with 2% HEPES (1 M, PAA Laboratories), 1% sodium pyruvate (100 mM, Sigma-Aldrich), 1% non-essential amino acids (100x, Sigma), 10% foetal bovine serum (FBS, Gibco) and 1% L-glutamine (200 mM, Sigma-Aldrich). FaDu and CAL33 cells were maintained in Eagle’s minimum essential medium (EMEM, ATCC) with 10% FBS. All cells were cultured at 37 °C in 5% CO_2_/ 95% air. For hypoxic conditions, cells were transferred to an InvivO_2_ Baker hypoxic chamber (Bridgend, UK) using 5% CO_2_, and 95% nitrogen to modulate oxygen levels.

### Seahorse X-Fe 96 extracellular metabolic flux analysis

Mitochondrial function was measured using a Seahorse XFe 96 Analyzer and XF cell Mito stress Test (Agilent technology, USA). Cells were seeded at 1 × 10^4^ cells/well into XFe-96 well culture plates and incubated overnight for attachment. To allow hypoxic equilibrium prior to treatment, cells were acclimatised at the required oxygen concentration for a minimum of 1 h before further treatment. Typically, cells were then treated with atovaquone (15, 30 µM) for 5 h before assay progression. After treatment, cells were washed with pre-warmed Seahorse XF media, and mitochondrial function assessed using sequential injections of 20 µL of 2.5 µM oligomycin (ATP synthase inhibitor), 22 µL of 2.0 µM FCCP (carbonylcyanide p-trifluoromethoxy phenylhydrazone), and 25 µL of 5 µM rotenone (complex I inhibitor)/antimycin (complex III inhibitor) in XF assay media. The XF Cell Mito Stress Test measured oxygen consumption rate (OCR - pM O_2_ consumption/min) and extracellular acidification rate (ECAR - change in pH units/min), assessing parameters such as basal and maximal respiration, and ATP-linked OCR.

### Radiation clonogenic assay

Clonogenic assays were conducted as previously described [24]. Briefly, 4 × 10^5^ cells were plated in 35 mm [2] dishes and allowed to adhere for 24 h. Cells were treated with atovaquone for 5 h, then irradiated with a single dose of 2, 4, 6, or 8 Gy. For hypoxic studies, cells were seeded, allowed to attach overnight, then transferred to a hypoxic station (InvivO_2_ Baker) with 0.5% O_2_/5% CO_2_, 95% N_2,_ for 4 h. Cells were then treated with atovaquone (30 µM) for 5 h and irradiated under hypoxic conditions in an airtight bag gassed with 0.5% O_2_/5% CO_2_, 95% N_2_. Post-radiation, cells were washed twice in PBS, detached, counted, reseeded at low densities under atmospheric O_2_, and incubated for 9–14 days to form colonies. Colonies were stained with 0.4% crystal violet in 70% methanol. Colonies with more than 50 cells were counted to determine plating efficiency (PE), defined as the number of colonies formed divided by the number of cells seeded. Surviving fractions (SF) were calculated relative to non-irradiated controls and fitted to the linear quadratic (LQ) model (Eq. 1).

1 $$\text{Linear quadratic:}\,\,\text{S}=\text{exp}(-\alpha\text{D}-\beta\text{D}^2)$$

LQ fits were calculated using least-squares regression in Prism 9.0 (GraphPad Software, CA, USA). The area under the curve (AUC) represents the mean inactivation dose (MID), which is used to calculate the sensitiser enhancement ratio (SER) by dividing the MID of untreated controls by atovaquone-treated cells. α and β components derived from the LQ fit, represent direct (α) and indirect (β) cell damage.

### Reactive oxygen species (ROS) level

ROS levels were measured using the MitoPY1 probe, which selectively associates with mitochondria of live cells, and responds to increased ROS flux via fluorescence activation. FaDu, CAL27 and CAL33 cells were seeded at 8 × 10^5^ cells/well in a 6-well plate and allowed to adhere overnight. For hypoxic cultures, cells were transferred to a hypoxic workstation (0.5% O_2_) for 4 h. Cells were then treated with MitoPY1 (10 µM) and atovaquone (30 µM) for 2 h under either normoxia (21% O_2_) or hypoxia (0.5% O_2_). Following irradiation with 4 Gy (Faxtrion CP-160 Arizona, USA), cells were returned to the incubator for a further 3 h. Cells were harvested by trypsinisation, centrifuged at 500 g for 5 min and resuspended in 400 µL of ice-cold assay buffer. Samples were analysed on FACSCalibur (BD Biosciences) and fluorescence was quantified using the geometric mean.

### Apoptosis assay

CAL27 cells were plated in 6-well plates at a density 2 × 10^5^ cells/well and allow to attach for 24 h. Cells were then exposed to hypoxia (0.5% O_2_) for 1 h, and treated with fresh medium containing atovaquone (30 µM), 3-methyladeine (3-MA, 2 mM) or the combination of 3-MA and atovaquone for a further 5 h prior to irradiation (6 Gy). Apoptosis assays were performed 24 h after radiation using the Annexin V-FITC Apoptosis Detection kit (BD Biosciences). Cells were harvested and washed twice with cold PBS, then suspended in 195 µL binding buffer at a density of 5 × 10^5^ cells/mL. After 10 min incubation with 5 µL of Annexin V-FITC, cells were resuspended in 190 µL binding buffer and 10 µL of propidium iodide. After a 15 min incubation at room temperature, flow cytometry was performed (BD FACSCalibur), with data analysis conducted using BD FACSuite TM software.

### RNA interference

siRNA transfections were carried out in Opti-MEM (Gibco) using lipofectamine RNAiMAX and 25 nM siRNA. Small interfering RNA against eIF2α and non-targeting scrambled siRNAs were purchased from Thermo Fisher Scientific. FaDu, or CAL27 cells were seeded at 2 × 10^5^ cells per well in 6-well dish. The siRNA sequences (5’-3’) were: sense strand - CACCUUCAUUUGUUAGAUUTT, antisense - AAUCUAAAUGAAGGUGCA. Transfection complexes were added in a dropwise manner and incubated for 24–48 h.

### Western blot analysis

Cells exposed to various treatment conditions were harvested and lysed in 1X radioimmunoprecipitation assay (RIPA) buffer with protease and phosphatase inhibitors [25]. 30 µg of protein was loaded per well and electrophoresed through a 4–12% sodium dodecyl-polyacrylamide gel (SDS-PAGE). Proteins were then transferred to a nitrocellulose membrane, blocked in 5% skim milk/PBST buffer for 1 h, and incubated at 4 °C overnight with the primary antibody (1:1000 dilution). After washing in TBST, HRP-conjugated secondary antibody (1:1000 dilution) was applied. Bands were visualised by chemiluminescence, imaged using a digital imaging system (UVI Tech, Cambridge, United Kingdom) and analysed using ImageJ. Semi-quantitative relative protein levels are expressed as a density ratio of the target protein against α-tubulin (housekeeping protein), then normalised against untreated controls. Raw images of individual western blot replicates are provided in the supplementary data (Supplementary Figure S4).

### Statistical analysis

All statistical analysis was performed using Graph-Pad Prism 9 software. Data are expressed as mean ± SEM. For clonogenic assays, two-way ANOVA with a Tukey multiple comparison test was used to determine differences between survival assay groups. For other experiments, one-way ANOVA with Tukey multiple comparisons test was used. Data were considered significant when *p* ≤ 0.05.

## Results

### Atovaquone reduces oxidative respiration in HPV (-) HNSCC cells

**Fig. 1:**
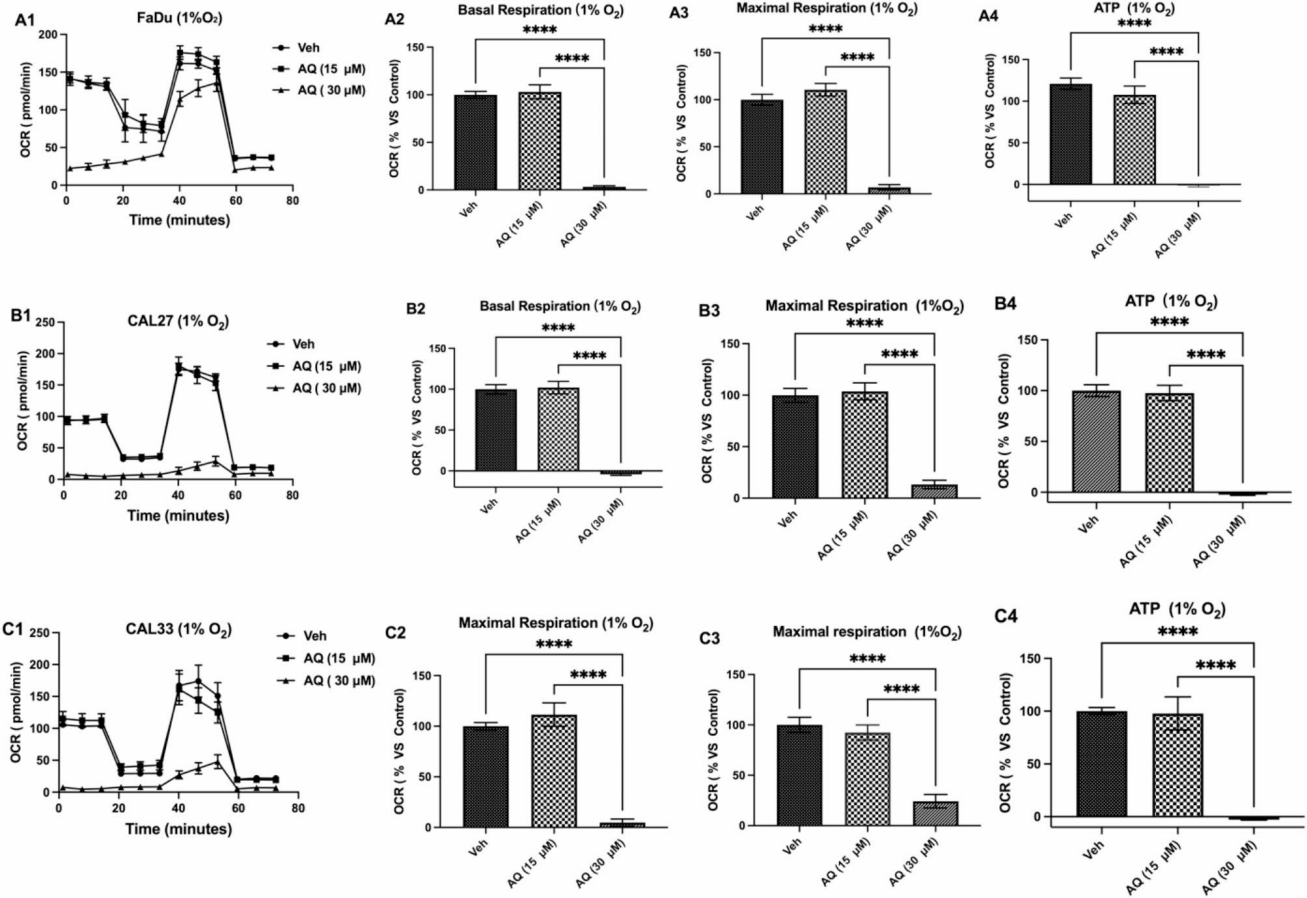
Atovaquone treatment inhibits mitochondrial respiration of HNSCC cells under hypoxia. The metabolic profile of hypoxic: **A1)** FaDu, **B1)** CAL27 and **C1)** CAL33 cells treated with atovaquone (15 µM or 30µM) for 5 h was then analysed using the Seahorse XFe96 Analyser. Significant reductions in **A-C2)** basal respiration, **A-C3)** maximal respiration and **A-C4)** ATP levels were observed. Data represents mean +/- SEM of *n* = 3 independent experiments. Statistical differences were determined using one-way ANOVA with a Tukey multiple comparison test, with **** *p* < 0.0001

The direct toxicity of atovaquone was assessed by Alamar blue assay with CAL27 and CAL33 IC_50_ concentrations exceeding 100 µM (FaDu– not reached– Figure S1 A-C). As such all subsequent experiments were conducted using a maximum non-toxic drug concentration of 30 µM. Atovaquone significantly inhibited mitochondrial respiration in all cell lines under both hypoxic (1% O_2_– Fig. 1A1-C1) and atmospheric (21% O_2_) oxygen levels (Figure S2 A-C), reducing basal respiration, maximal respiration, and ATP production. More specifically, in FaDu cells, 15 µM atovaquone failed to significantly impact basal and maximal respiration, along with ATP production, however, increasing the drug concentration to 30 µM significantly reduced all three measurements (Fig. 1. A2-A4): basal respiration (*p* < 0.0001); maximal respiration (*p* < 0.0001); ATP production (*p* < 0.0001). Similar magnitude effects were also observed in CAL27 and CAL33 (Fig. 1B&C2-4). Under normoxic conditions comparable trends were observed across all three cell lines, although the magnitude of effect was slightly tempered reducing FaDu basal respiration (*p* < 0.0001), maximal respiration (*p* < 0.0001) and ATP production by 73%, 50.3% and 80.3% respectively (Figure S2 A2& A3).

Interestingly, under hypoxia OXPHOS inhibition by atovaquone (30 µM) forced a significant increase in glycolytic dependence, increasing the basal extracellular acidification rate (ECAR) by 69%, 79% for 49% in FaDu (*p* = 0.036), CAL27 (*p* < 0.001) and CAL33 cells (*p* = 0.009) respectively (Figure S3). The full extent by which atovaquone drives a switch in energy production from oxygen dependent ATP production to glycolysis is neatly illustrated by plotting OCR against ECAR, yielding an energy map that irrespective of tumour cell model consistently points to rapid and significant metabolic adaption, responses that were observed under both hypoxic (Figure S3 A-C3) and normoxic (Figure S4 A-C3) environmental conditions.

### Atovaquone mediated radiosensitisation

**Fig. 2:**
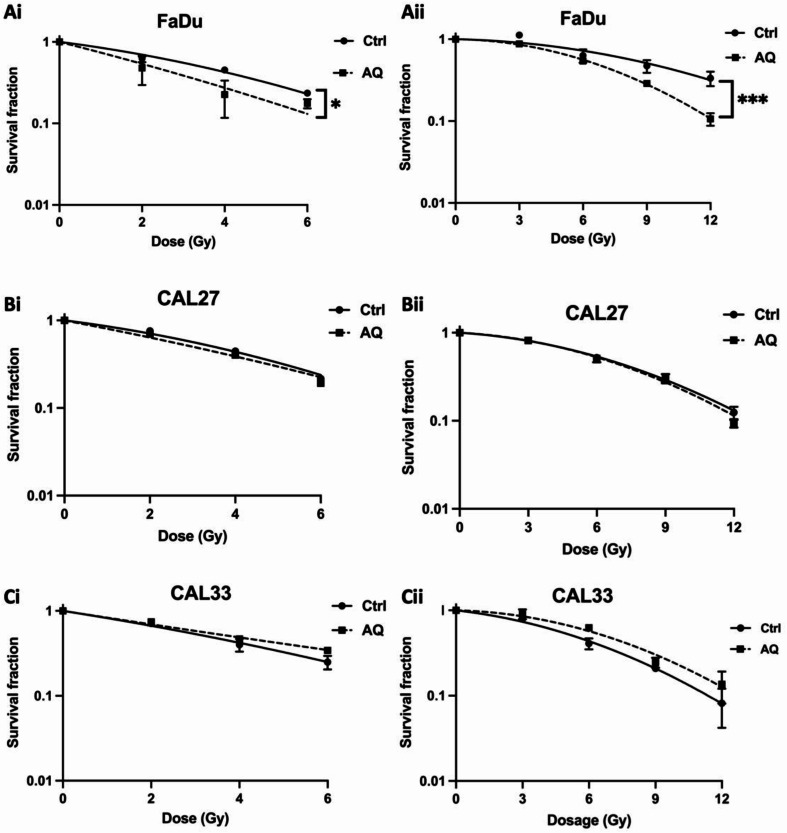
Atovaquone selectively radiosensitises normoxic and hypoxic tumour cells. Clonogenic radiation survival curves for **(A)** FaDu, **(B)** CAL27 and **(C)** CAL33 cells treated with atovaquone (30 µM) under both **(i)** normoxic (21% O_2_) and **(ii)** hypoxic (0.5% O_2_) conditions. Data represents mean +/- SEM of *n* = 3 independent experiments. Statistical differences determined was a two-way ANOVA with a Tukey multiple comparison test, with * representing *p* < 0.05 and ***p < 0.001

Several studies have shown that inhibiting intracellular oxygen consumption increases radiation sensitivity [26, 27]. Therefore, we tested whether atovaquone could sensitise hypoxic/normoxic tumour cells to radiation using the colony forming assay. Atovaquone (30 µM) significantly (*p* < 0.05) increased normoxic FaDu radiosensitivity, yielding a sensitiser enhancement ratio (SER) of 1.43 (Fig. 2Ai). Hypoxia (0.5% O_2_) increased radiation resistance by ~ 2-fold (oxygen enhancement ratio OER-2.1), indicating maintenance of hypoxic conditions during radiation treatment (Table S1). Despite this, atovaquone acted as a potent hypoxic radiosensitiser (*p* < 0.001) of FaDu cells generating a SER of 1.34 (Fig. 2Aii). Surprisingly, atovaquone produced different intrinsic radiosensitising effects depending on cell line, failing to modulate the radiation response in CAL27 (Fig. 2Bi&ii) or CAL33 (Fig. 2Ci&ii) under either normoxic or hypoxic conditions.

### Atovaquone induced production of mitochondrial reactive oxygen species (mROS)

**Fig. 3:**
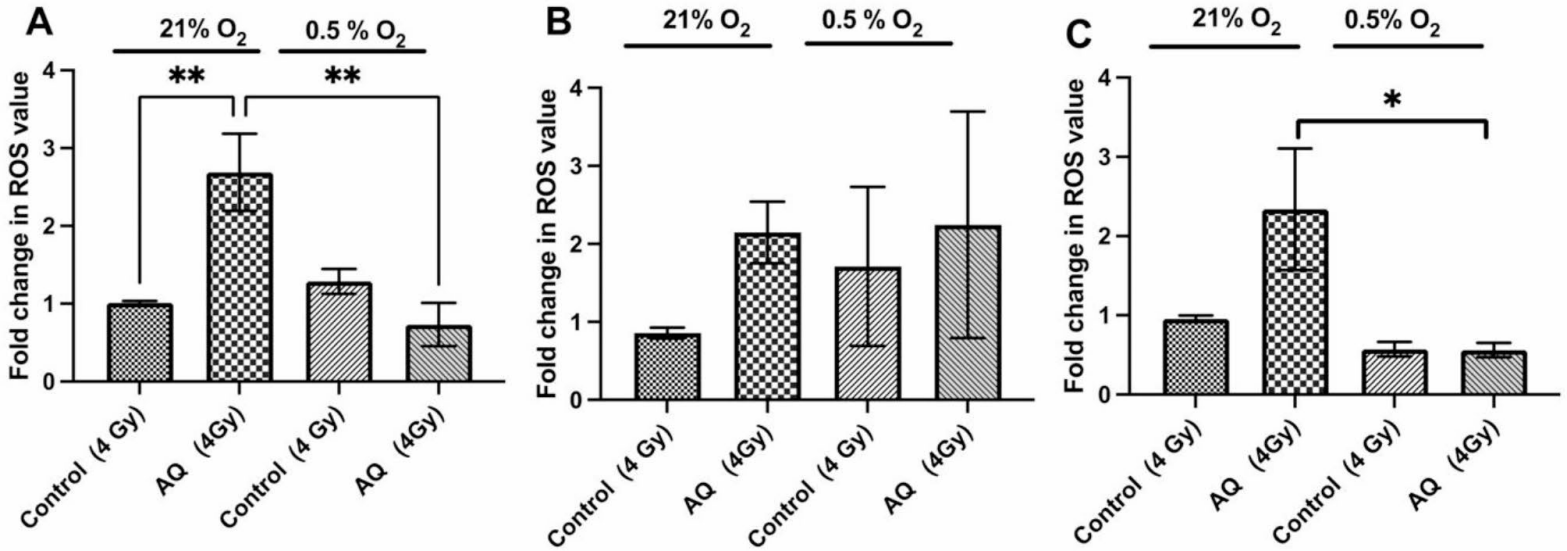
Atovaquone plus radiation treatment induces mROS in HNSCC cells. ROS level of **(A)** FaDu, **(B)** CAL27 and **(C)** CAL33 cells treated radiation alone (4 Gy) or atovaquone plus radiation under normoxic (21% O_2_) or hypoxic (0.5% O_2_) conditions. Data represents mean +/- SEM of *n* = 3 independent experiments. Statistical differences were determined using one-way ANOVA with a Tukey multiple comparison test, with * representing *p* < 0.05 and ** *p* < 0.01

Mitochondrial electron transport chain (mt-ETC) complexes (I and III) are major sources of intracellular reactive oxygen species (ROS). To investigate if atovaquone-mediated radiosensitisation is partly due to increased oxidative stress, we evaluated ROS generation using MitoPY1, a probe specific for mitochondrial hydrogen peroxide (H_2_O_2_). Our results reveal that combining radiation with atovaquone significantly (*p* < 0.01) increases H_2_O_2_ production in FaDu cells (2.68-fold), with similar trends in CAL27 (2.39-fold) and CAL33 (2.44-fold) cells compared to radiation alone under atmospheric oxygen (Fig. 3A-C). However, under hypoxic conditions, the effect of atovaquone on ETC-derived H_2_O_2_ was reduced, with a significant drop (~ 75%) in FaDu (*p* < 0.01) and CAL33 (*p* < 0.05) cells compared to normoxia.

### Atovaquone activates the ISR by increasing eIF2α phosphorylation in HNSCC cells

**Fig. 4:**
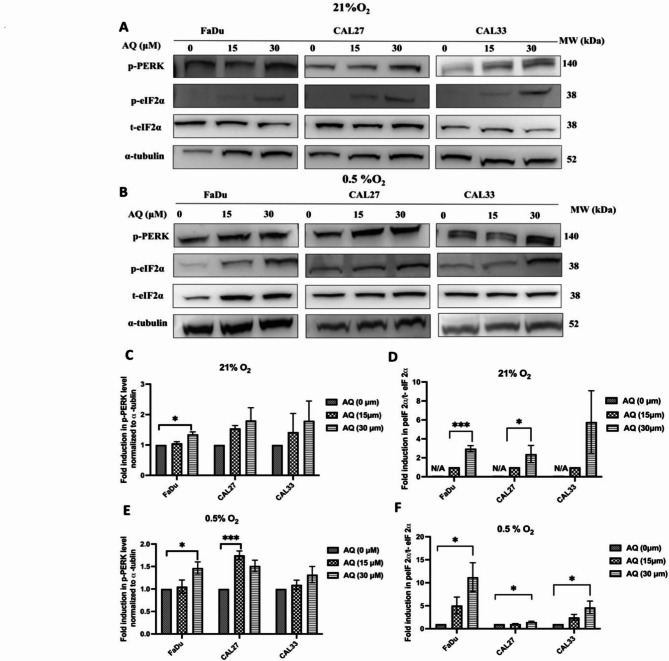
Atovaquone activates ISR signaling via increased eIF2α phosphorylation. FaDu, CAL27 and CAL33 cells were treated with atovaquone (30 µM M) for 5 h under **(A)** atmospheric oxygen (21% O_2_) or **(B)** hypoxia (0.5% O_2_), then analysed by western blot for alterations in p-PERK, p-eIF2α, t-eIF2α and α-tubulin, using the latter as the loading control. Quantitative densitometric analysis for: **C&D)** p-PERK and p-eIF2α under normoxic (21% O_2_) and **E&F)** hypoxic (0.5% O_2_) conditions. Blots and quantified data are representative of the mean of a minimum of three independent experiments ± SEM. Statistical differences were determined using one-way ANOVA with a Tukey multiple comparison test, with * representing *p* ≤ 0.05, *** *p* ≤ 0.001

To explore the mechanisms underpinning the variable radiosensitising properties of atovaquone, we next investigated the role of ISR signaling, known to be activated by mitochondrial dysfunction. We hypothesised that atovaquone might induce the ISR response, promoting eIF2α phosphorylation and contributing to radiation resistance under hypoxia. Of the four eIF2α activating kinases we excluded studies into PKR, given that the phosphorylation activity of this kinase is primarily activated by viral double-stranded (ds) RNA molecules. HRI, PERK and GNC2 are respectively activated by oxidative stress, endoplasmic reticulum stress and amino acid [28]. Atovaquone (30 µM) significantly (*p* < 0.05) increased normoxic PERK phosphorylation by ~ 30% in FaDu cells, with similar magnitude effects in CAL27 and CAL33 (Figs. 4A&C), leading to downstream phosphorylation of eIF2α, proving significant in FaDu (1.96-fold– *p* < 0.001) and CAL27 cells (1.39-fold– *p* < 0.05) (Fig. 4A&D). Under hypoxia, 30 µM atovaquone again significantly (*p* < 0.05) increased PERK phosphorylation by ~ 46% in FaDu cells, with similar results in CAL27 and CAL33 cells (Figs. 4B&E), leading to marked eIF2α phosphorylation compared to untreated controls (FaDu 11.2-fold; CAL27 1.5-fold; CAL33 4.6-fold - Figs. 4B&F). Surprisingly, considering the role of HRI in sensing oxidative stress, atovaquone had no direct impact on HRI expression or activation of p-GNC2 (not detected), irrespective of normoxic or hypoxic exposure conditions (Figure S5). These data suggest that eIF2α phosphorylation is primarily regulated by ER stress mediated UPR activation.

### Atovaquone induces G_1_ phase block in HNSCC cells

**Fig. 5:**
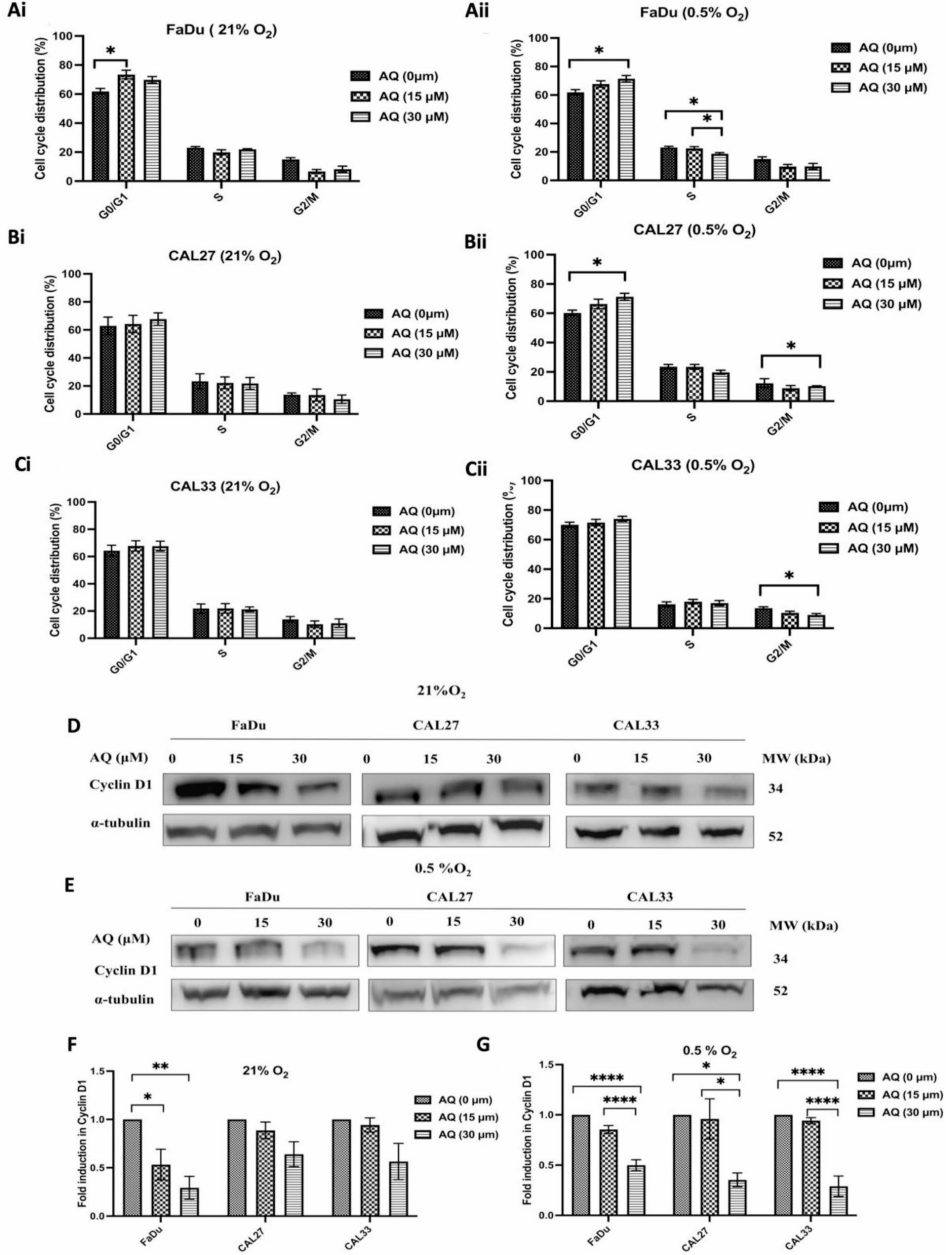
Atovaquone induces G_1_ accumulation by suppressing translation of cyclin D_1_. **(A)** FaDu, **(B)** CAL27 and **(C)** CAL33 cells were treated with atovaquone (15 or 30 µM) for 5 h under **(i)** normoxic (21% O_2_) or **(ii)** hypoxic (0.5% O_2_) with data representing the distribution of G_0_/G_1_, S and G_2_/M phase determined by flow cytometry. **D&E)** Normoxic and hypoxic cyclin D_1_ protein expression following atovaquone treatment as determined by western blot. **F&G)** Quantitative analysis of cyclin D_1_ protein expression normalised against α-tubulin. Quantified data and western blots are representative of the mean of a minimum of three independent experiments ± SEM. Statistical differences were determined using one-way ANOVA with a Tukey multiple comparison test, with * representing *p* ≤ 0.05, ** *p* ≤ 0.01 and ****p < 0.0001

Previous studies showed that ISR activation can arrest cell cycle progression by reducing cyclin D_1_ expression, leading to G_1_/G_0_ arrest [29, 30]. Cell cycle affects radiosensitivity, with G_2_/M cells proving more sensitive, whereas G_0_/G_1_ cells are comparatively resistant [31], linking ISR-mediated arrest with radioresistance. We investigated whether atovaquone induces cell cycle arrest through cyclin D_1_ deregulation under normoxic and hypoxic conditions. Under normoxia, atovaquone significantly (*p* = 0.0146) increased FaDu cells in G_0_/G_1_ from 61.9 to 73.4% (Fig. 5Ai), an effect not observed in CAL27 and CAL33 cells (Figs. 5 Bi&Ci). Hypoxia amplified this effect, with atovaquone significantly (*p* = 0.0221) increasing the FaDu G_0_/G_1_ fraction from 61.8 to 71.4%, while decreasing S phase cells from 23.1 to 18.7% (Fig. 5 Aii - *p* = 0.0156). Atovaquone also induced G_0_/G_1_ accumulation in CAL27 cells, reducing G_2_/M cells from 17.1 to 7.9% (Fig. 5 Bii - *p* = 0.0049). Further support of atovaquone mediated cell cycle effects were obtained by western blot, showing a significant (*p* < 0.01) 71% reduction in cyclin D_1_ levels under normoxia in FaDu cells, with similar, though less pronounced, effects in CAL27 and CAL33 cells (Fig. 5D&E). Hypoxia further enhanced these effects, with atovaquone reducing cyclin D_1_ by 50% (*p* < 0.0001), 65% (*p* < 0.05), and 71% (*p* < 0.0001) in FaDu, CAL27, and CAL33 cells, respectively (Fig. 5F&G).

### Atovaquone activation of ISR alters the genomic response to hypoxia

**Fig. 6:**
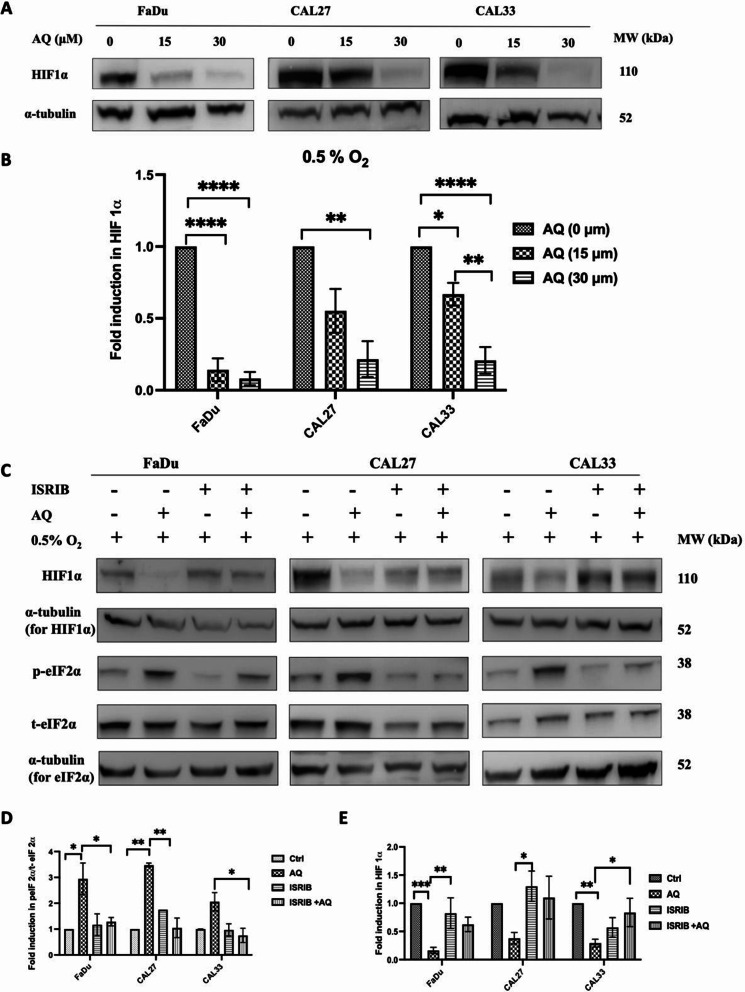
Atovaquone treatment reduces HIF-1α translation via activation of ISR. ISR suppresses hypoxia-dependent HIF-1α stablisation in an eIF2α dependent manner. **(A)** Representative western blots of HIF-1α protein expression in FaDu, CAL27 and CAL33 cells treated with atovaquone (15/30 µM) then exposed to hypoxia (0.5% O_2_) for 5 h. **(B)** Quantitative analysis of HIF-1α protein levels normalised against α-tubulin used as a loading control. **(C)** Representative western blots of HIF-1α, t-eIF2α, and p-eIF2α expression in FaDu, CAL27 and CAL33 cells treated with atovaquone (30 µM) and exposed to 0.5% O_2_ for 5 h. Where indicated cells were co-treated with ISRIB (1 µM), an eIF2α inhibitor. **(D)** Quantitative analysis of the ratio of p-eIF2α against t-eIF2α normalised against α-tubulin used as a loading control. **(E)** Quantitative analysis of HIF-1α protein levels normalised against α-tubulin used as a loading control. Data are representative of the mean of three independent experiments ± SEM. Statistical differences were determined using one-way ANOVA with a Tukey multiple comparison test, with * representing *p* ≤ 0.05, **p ≤ 0.01, ***p < 0.001 and ****p ≤ 0.0001

Unsurprisingly, hypoxia (0.5% O_2_) induced HIF-1α expression in all three tumour cell models. However, atovaquone (30 µM) potently attenuated HIF-1α levels by 85% in FaDu cells, and by 78% and 79% in CAL27 and CAL33 cells, respectively (Figs. 6 A&B).

As demonstrated in Fig. 4B, atovaquone increased phosphorylation of eIF2α, an effect hypothesised to promote ISR mediated translational repression [32]. To link increased eIF2α activation to atovaquone induced HIF-1α suppression, we next pre-treated cells with ISRIB, a selective inhibitor of the integrated stress response (ISR). ISRIB selectively and potently reverses eIF2α phosphorylation, restoring ISR mediated translational repression, without impacting non-stressed cells. Unsurprisingly, in the absence of atovaquone, IRSB had no direct impact on eIF2α phosphorylation, while atovaquone alone significantly (FaDu and CAL33 *p* < 0.05; CAL27 *p* < 0.01) increased eIF2α levels by 2-3 fold (Fig. 6C&D). However, pre-treatment of IRSB combined with atovaquone potently supresses eIF2α phosphorylation and the subsequent ISR response, restoring HIF-1α stabilisation (Fig. 6C&E). Interestingly, this response was common to all three tumour cell models, indicating that atovaquone-mediated HIF-1α downregulation is largely eIF2α-dependent (Fig. 6C&D).

To probe this response further we also examined how atovaquone-modulated ISR impacts expression of key HIF targeted genes (Figure S6). As expected, significant increase in PDK1 expression was observed following combined ISRIB and atovaquone treatment, especially in CAL27 and CAL33 cells (Figure S6 B&C). Similar, though less significant, effects were also seen with CAIX-1, increased by hypoxia and suppressed by atovaquone, with ISRIB/atovaquone combination rescuing the expected CAIX-1 response to hypoxia (Figure S6 D-F).

### Atovaquone regulation of autophagy and apoptosis

**Fig. 7:**
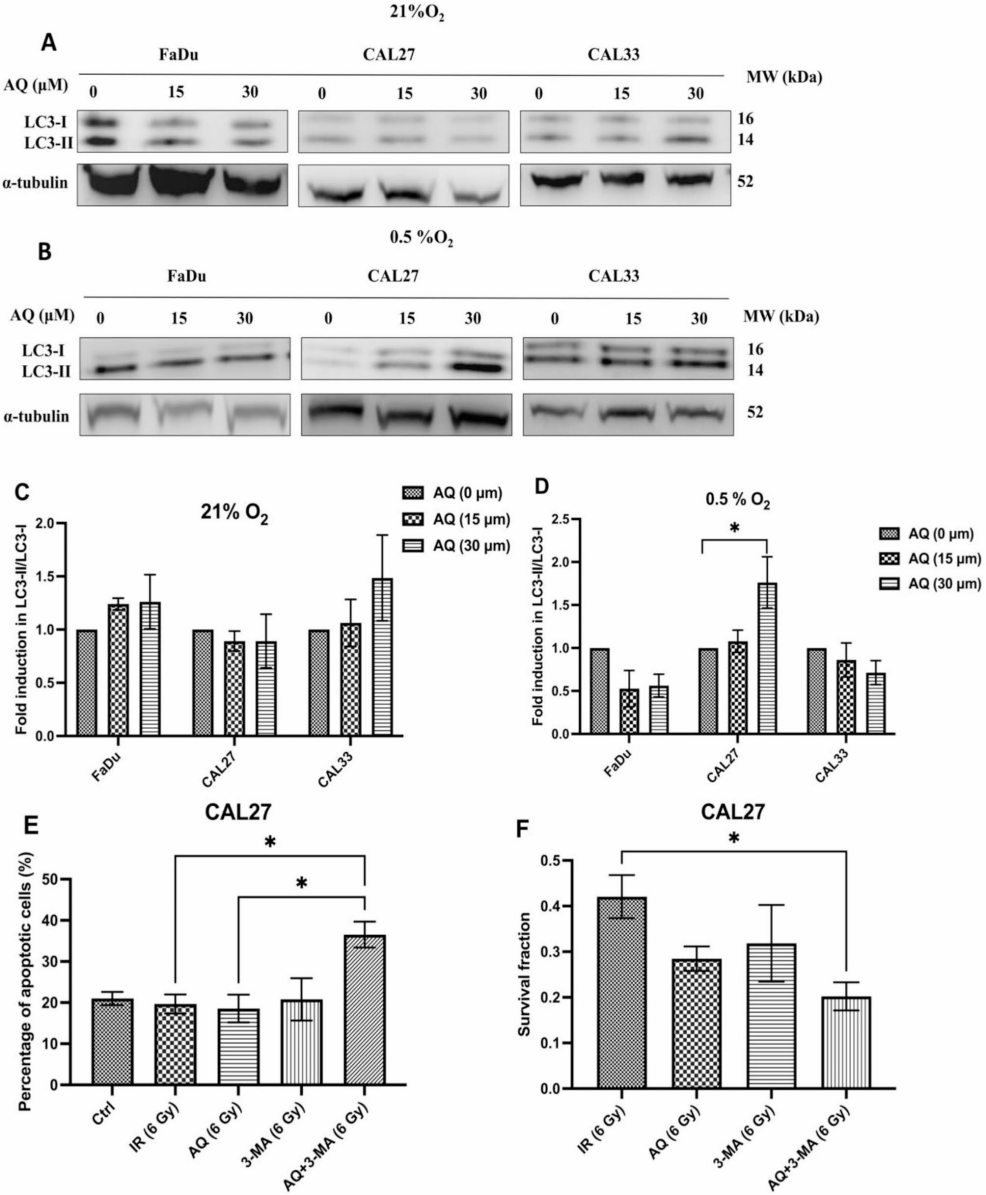
Autophagy mitigates the radiosensitising effects of atovaquone under hypoxia Atovaquone selectively promoted autophagy in hypoxic CAL27 cells, promoting resistance to atovaquone under hypoxia. Representative western blots of relative LC3-I and LC3-II expression in **(A)** normoxic and **(B)** hypoxic atovaquone treated FaDu, CAL27 and CAL33 cells. **C&D)** Quantified densitometric anlayis of the relative LC3II/LC3-I ratio normialised against the α-tubulin used as a loading control. **E)** Annexin-V/PI flow cytometry analysis of apoptosis following autophagy inhibition with 3-MA (2 mM), combined with atovaqoune and radiation under hypoxia (0.5% O_2_). **F)** Autophagy inhibition by 3-MA combined with atovaquone sensitisies hypoxic CAL27 to radiation (6 Gy). Data are representative of the mean of three independent experiments ± SEM. Statistical differences were determined using one-way ANOVA with a Tukey multiple comparison test, with * representing *p* ≤ 0.05

ISR activation is linked with autophagy induction under metabolic stress, potentially increasing radiation resistance [33–35]. Observing that atovaquone activates the ISR, we next examined the effect of atovaquone on autophagy. Under normoxia, atovaquone did not alter LC3-II levels, a marker of autophagy, and therefore under these conditions does not promote an autophagic response (Figs. 7A&C). Under hypoxia, atovaquone significantly (*p* < 0.05) increased the conversion of LC3-I to LC3-II by 1.8-fold in CAL27 cells (Figs. 7B&D).

Since autophagy is associated with increased hypoxia-induced radioresistance, we explored whether autophagy inhibition might enhance apoptosis and radiation sensitivity, selecting CAL27 as the test model due to the evidence for autophagy in these cells. We used 3-methyladenine (3-MA), an inhibitor of autophagosome formation. Our data show that 3-MA combined with atovaquone significantly (*p* < 0.05) increased apoptosis and radiosensitivity, as indicated by Annexin V/PI staining (Fig. 7E and Figure S7) and reduced colony formation (Fig. 7F). Within 24 h post treatment, radiation alone did not influence the apoptotic fraction, an unsurprising effect due to delayed radiation induced cell death, predominantly caused by mitotic catastrophe. Similarly, atovaquone or 3-MA combined with radiation had no direct impact on apoptotic fraction. However, the combination of atovaquone, 3-MA, and radiation doubled the apoptotic fraction from 18 to 36% (*p* < 0.05), indicating that suppressed autophagy promotes cell death (Fig. 7E). This finding was confirmed by the increased sensitivity to a single 6 Gy fraction, with approximately a 33% reduction in clonogenicity compared to cells treated with radiation plus either atovaquone or 3-MA alone. Together, these results demonstrate that atovaquone-induced autophagy in CAL27 cells acts as a pro-survival mechanism, with autophagy inhibition enhancing atovaquone radiosensitisation.

### ISR inhibition renders HNSCC tumour models susceptible to atovaquone mediated radiosensitisation

**Fig. 8:**
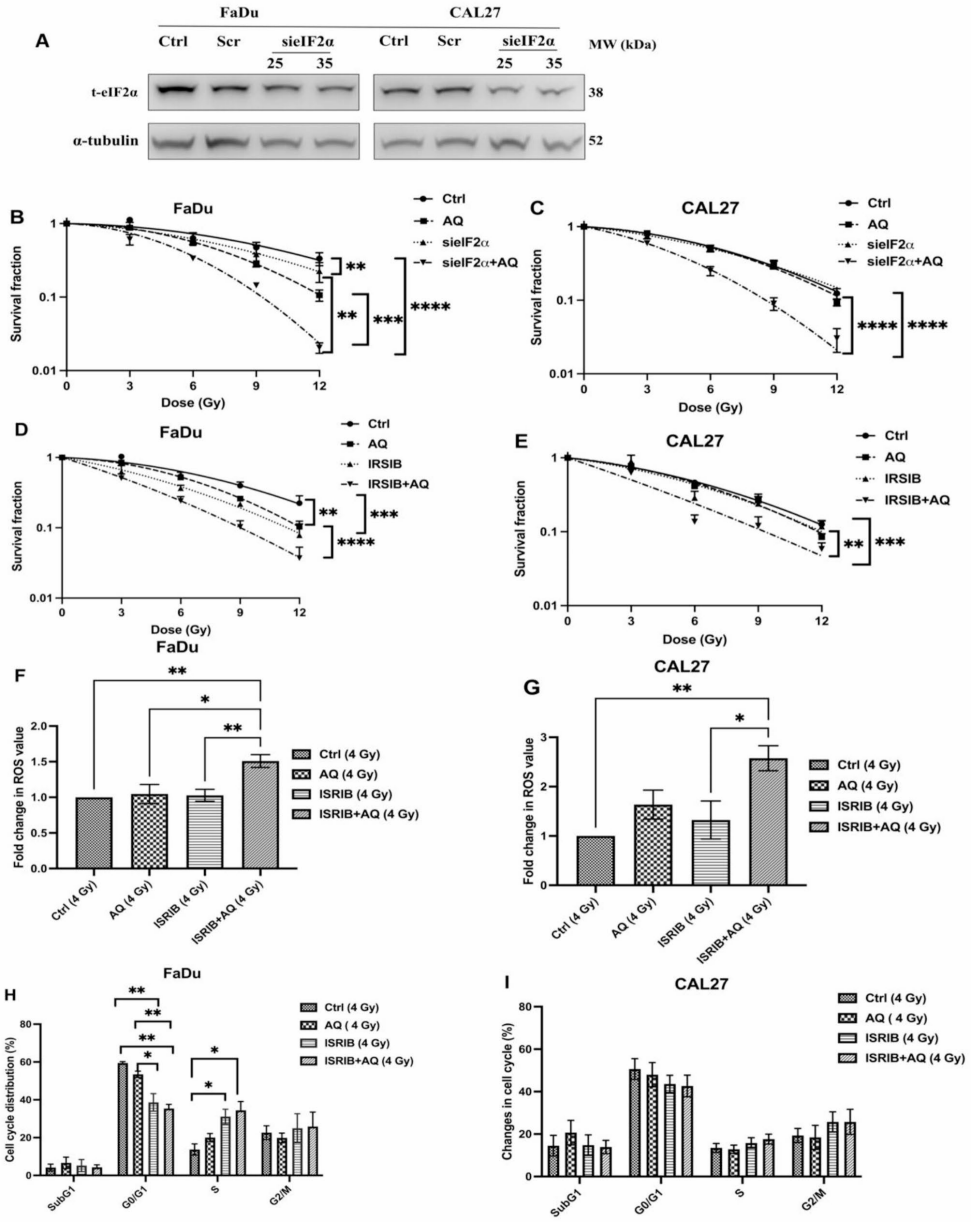
Atovaquone consistantly radiosensitises hypoxic tumour cells following eIF2α downregulation or pharmacological inhibition. **(A)** Representative western blots confirming RNAi (25 & 35 nM) supression of eIF2α maintained for at least 48 h. Hypoxic clongenic radiation survival curves following eIF2α knockdown and atovaquone treatment in **(B)** FaDu and **(C)** CAL27 cells. Hypoxic clongenic radiation survival curves following eIF2α inhibition with ISRIB (1µM) and atovaquone treatment in **(D)** FaDu and **(E)** CAL27 cells. Intracellular H_2_O_2_ levels detected using MitoPY1 in irridiated (4 Gy), hypoxic (0.5% O_2_) **(F)** FaDu and **(G)** CAL27 cells following treatment with atovaquone, ISRIB or the combination. Cell cycle distribution of irridiated (4 Gy) hypoxic (0.5% O_2_) **(H)** FaDu and **(I)** CAL27 cells after treatment with atovaquone, ISRIB or the combination. Data are representative of the mean of three independent experiments ± SEM. Statistical differences were determined using one-way ANOVA with a Tukey multiple comparison test, with * representing *p* < 0.05, **p ≤ 0.01, ***p < 0.001 and ****p ≤ 0.0001

To confirm the role of ISR signaling in atovaquone-mediated radioresistance, eIF2α, the downstream effector, was knocked down (KD) using siRNA. Treatment with si-eIF2α (35 nM) reduced total eIF2α levels by 45% and 61% in FaDu and CAL27 cells, respectively (Fig. 8A). Control scrambled siRNA did not alter eIF2α expression.

Using these siRNA KD models, clonogenic assays confirmed the role of ISR in suppressing the radiosensitising potential of atovaquone. Under hypoxia, eIF2α knockdown alone had no impact on radiation response in FaDu or CAL27 cells (Fig. 8B&C). Similar to Fig. 2 Aii, atovaquone significantly increased FaDu radiosensitivity, generating an SER value of 1.34. This response was significantly (*p* < 0.001) amplified with eIF2α KD, generating an SER of 1.99, nearly ablating hypoxia-induced radioresistance (Fig. 8B). In CAL27 cells, atovaquone alone had no radiosensitising effect, mirroring Fig. 2Bii. However, eIF2α KD combined with atovaquone significantly (*p* < 0.01) increased radiation sensitivity, yielding an SER of 1.59 (Fig. 8C).

To ensure these effects were not off-target siRNA effects and to assess potential therapeutic value, we evaluated pharmacological eIF2α inhibition using ISRIB. Unlike eIF2α siRNA, ISRIB treatment of FaDu cells contributed to radiation sensitivity under hypoxia, yielding a mean SER of 1.61. Recapitulating the effect seen with siRNA, ISRIB plus atovaquone almost doubled radiation sensitivity over ISRIB, producing a highly significant (*p* < 0.0001) SER increase to 2.12 (Fig. 8D). In CAL27 cells, ISRIB or atovaquone alone did not alter radiation response. However, combined atovaquone and ISRIB significantly reversed hypoxia-induced resistance (*p* = 0.0027), producing a mean SER of 1.59 (Fig. 8E).

Under these hypoxic radiosensitising conditions we again assayed mitochondrial ROS levels using MitoPY1. Atovaquone alone did not significantly increase mROS, corroborating our earlier observation. However, combined eIF2α inhibition and atovaquone significantly (*p* = 0.0128) elevated H_2_O_2_ levels by 30% over those with atovaquone treatment alone (Fig. 8F), with a similar response equating to a 2.5-fold increase (*p* = 0.004) over radiation-only treated CAL27 cells (Fig. 8G).

With ISR activation inducing cell cycle dormancy and promoting survival under stress, re-analysis of cell cycle distribution with combined ISR/ETC inhibition was conducted. Combined ISR inhibition with atovaquone significantly (*p* = 0.0029) suppressed the G_1_ fraction compared to radiation alone, reducing it by 25%, with a concurrent 16% increase in the S phase fraction following ISRIB treatment (Fig. 8H). Similar trends, though non-significant, were also seen in CAL27 cells (Fig. 8I).

Together, these data demonstrate that inhibiting ISR signaling promotes atovaquone mediated radiosensitisation, especially in models with no direct radiosensitising effect from atovaquone alone.

## Discussion

Robust pre-clinical studies have proven the ability of repurposed atovaquone to act as a potent metabolic radiosensitiser [12], results that have led to the commencement of several clinical studies (NCT02628080; NCT04648033). However, in our hands, the magnitude of effect varied considerably between tumour cell models of HPV(-) HNSCC, a response that led us to explore the underpinning adaptive mechanisms that limited the response to OXPHOS inhibition [36]. Herein, we found that atovaquone effectively reduces mitochondrial respiration, inducing endoplasmic reticulum stress and activating the ISR signaling, protecting against drug-induced radiosensitisation.

Atovaquone suppressed mitochondrial respiration across all tumour models tested, reducing basal and maximal respiration as well as ATP production (Fig. 1, Figure S2), with the strongest effects observed under hypoxic stress. This response could be attributed to compromised respiratory capacity [37], however, it is more likely that complex III inhibition forces increased reliance on glycolytic metabolism, an effect consistent with previous reports [38], and our data demonstrating elevated extracellular acidification [39]. This adaptation helps maintain intracellular ATP levels when OXPHOS is impaired, a response also observed with other OXPHOS inhibitors such as metformin in mice and patients [40]. “Metabolic radiosensitisation” is a concept that exploits the demand for oxygen by suppressing mitochondrial oxidative metabolism. Previous work demonstrated that reducing oxygen consumption alleviates hypoxia, helping overcoming hypoxia induced radioresistance [12]. While atovaquone increased radiosensitivity in FaDu cells, it did not overcome hypoxia-induced radioresistance in CAL27 and CAL33 models, suggesting the existence of an adaptive survival response under stress.

ISR is conservative mechanism sustaining homeostasis in response to stress. Depending on cell type, stress duration and intensity, among other factors, cells exhibit differential outcomes following ISR activation. Recent reports demonstrate that ISR signaling promotes survival under nutrient deprivation and hypoxia [41]. To the best of our knowledge, we found no prior reports linking atovaquone to ISR activation and radiation resistance. Initiating the ISR requires activation of one or more kinases responsible for eIF2α phosphorylation, including HRI, GCN2, PKR and HRI each conveying phosphorylation activity on a unique eIF2α serine residue [42]. Under hypoxic stress the ISR serves as an important pro-survival mechanism with the eIF2α/ATF4 axis proving crucial for hypoxic cell survival and influencing radiation response [43]. Our data demonstrate that hypoxia triggers ISR signaling through eIF2α phosphorylation, a response further enhanced by atovaquone, consistent with previous studies showing that mitochondrial dysfunction leads to ISR activation [44, 45]. Uncertainly remains in relation to the exact mechanism, with some studies indicating that GCN2 activation is activated following ETC defects in a number of cells [44]. Additionally mitochondrial stress can also induce the ISR via mitochondrial protease OMA1 activity, with OMA1 cleaving a little known protein named DELE1, promoting cytoplasmic translocation, activating HRI [46, 47]. Interestingly, our data did not point to atovaquone mediated activation of either of these ISR kinases, predominantly indicating that effects were mediated though drug induced ER stress. Decreased ATP availability due to ETC inhibition also likely leads to ISR activation [48]. Our Seahorse data demonstrate atovaquone suppression of ATP under both hypoxia and normoxia, indicating that ETC inhibition activates ISR signaling. ATP depletion also activates AMPK through threonine-172 phosphorylation, promoting activity of a master metabolic regulator [49, 50]. Some studies suggest AMPK activation potentiates radiation-induced apoptosis, while defective mitochondria induce autophagy, promoting cell survival via AMPK signaling [51–54]. Herein, we found that atovaquone induces the ISR in HNSCC cell lines, possessing limited and cell specific radiosensitising effects under hypoxia, indicating that ISR stimulation may represent a novel mechanism conferring protection against atovaquone mediated radiosensitisation.

ISR-regulated cell cycle arrest is linked to radiation resistance [55, 56]. To establish if atovaquone is sufficient to cause cell cycle arrest, we analysed expression levels of cyclin D_1_, a central regulator of the cell cycle, showing potent drug induced cyclin D_1_ suppression. These findings support reports of decreased cyclin D_1_ reflecting wider attenuation of protein synthesis following ETC inhibition [57, 58]. Reduced global protein synthesis is linked to suppression of the key ribosomal subunit 80 S, mediated by eIF2α phosphorylation, driven by ER stress [59]. Hammanaka et al., (2005) also found that cyclin D_1_ repression is found to be dependent on eIF2α activation [60]. These studies demonstrate that eIF2α phosphorylation is a critical effector linking ER stress to cell cycle control. Our data show that atovaquone drives eIF2α activation and cyclin D1 depletion under hypoxia in all tested cell lines (Fig. 5E).

In our hands atovaquone consistently repressed HIF-1α, the key hypoxia-regulated transcription factor, supporting earlier reports that complex III inhibition is associated with oxygen sensing [61, 62]. Similar observations have been reported with complex I and complex V inhibitors, where treatment prevented HIF-1α stabilisation mediated by increased intracellular oxygen availability [63–65]. Bell et al. (2007) suggest that complex III Q_0_ is crucial for the transduction of hypoxic signals, releasing reactive oxygen species that stabilise HIF-1α, with subsequent genetic or pharmacological manipulation of complex III resulting in HIF-1α hydroxylation, preventing stabilisation and resulting in degradation [66]. Furthermore, eIF2α inhibitors are also associated with HIF-1α degradation [67], linking atovaquone mediated suppression of HIF-1α to UPR activation. More importantly, our data also demonstrated that activation of PERK/eIF2α suppresses hypoxia induced accumulation of HIF-1α and several HIF-1α dependent target genes (CAIX and PDK1). This is consistent with previous reports where PERK/eIF2α activation was shown to inhibit RNA-binding protein YB-1 from interacting with the 5’ untranslated region of HIF-1α mRNA, supressing HIF-1α translation, resulting in reduced HIF transcriptional activity [32].

Autophagy is a major catabolic process delivering proteins, cytoplasmic components, and organelles for lysosomal degradation. Autophagy is active in tumour cells, stimulated by intracellular stressors including mTOR inhibition, DNA damage, hypoxia and nutrient depletion [68]. ISR has been demonstrated to modulate cell survival and cell death pathways through the activation of autophagy and phosphorylation of eIF2α at S511, a step that appears essential to stress-induced autophagy [42]. The impact of ISR-triggered autophagy depends on tumour stage, inhibiting early-stage tumour progression but promoting survival and radiation resistance in advanced disease [19, 69, 70]. In this study, we report that atovaquone preferentially induces autophagy in CAL27 cells, antagonising atovaquone-induced apoptosis, contributing to radioresistance under hypoxic conditions.

As described above, ER stress can protect against extreme hypoxia, contributing to adaptive signaling that confers treatment resistance, including to radiotherapy [43, 71, 72]. We report that hypoxia triggers eIF2α phosphorylation, an effect enhanced by atovaquone. Interestingly, eIF2α suppression (siRNA or small molecule) enhances the radiosensitising potential of atovaquone, indicating eIF2α protects against the effects of atovaquone under hypoxia. Emerging evidence suggests that the induction of an antioxidant response, mediated though ISR activation, influences tumour cell radiosensitivity. The eIF2α/ATF4 axis is thought to confer protection by promoting glutathione (GSH) production [21]. Dephosphorylation of eIF2α increased IR induced ROS yields, disrupting ATF4-mediated glutathione biosynthesis, causing both in vitro and in vivo radiosensitisation [21]. Similar observations by Rouschop et al.. (2018) demonstrated that active eIF2α is involved in the survival of a subset of hypoxic tumour cells that determine radioresistance. Our work demonstrated that combined eIF2α inhibition and atovaquone promotes cell cycle redistribution and significantly enhances mitochondrial ROS production and compared to atovaquone alone, indicating that PERK/eIF2α activation following atovaquone treatment, and may play an important role in regulating resistance to IR.

In conclusion, our research reveals that atovaquone disrupts bioenergetics by imposing severe energy restrictions, which trigger widespread metabolic stress, activating ISR signaling through an eIF2α-dependent mechanism. This disruption can promote autophagy and confer resistance to atovaquone radiosensitisation. Notably, inhibiting autophagy or eIF2α, whether through siRNA or pharmacological means, can restore the radiosensitising effects of atovaquone by increasing ROS yields and reduction in G_0_/G_1_ arrest. While these experiments provide strong in vitro mechanistic data, the work could be complemented by assessing the systemic impact of atovaquone/radiation in a PERK/eIF2α defective setting, representing a limitation of the current work. These findings highlight the potential of targeting the PERK/eIF2α as a promising strategy to enhance metabolic radiosensitisation.

## Electronic supplementary material

Below is the link to the electronic supplementary material.

Supplementary Material 1

Supplementary Material 2

## Data Availability

No datasets were generated or analysed during the current study.
