# Supplementary material for: Atovaquone-induced activation of the PERK/eIF2α signaling axis mitigates metabolic radiosensitisation
Source: Cell Commun Signal. 2025 Apr 2;23:164. doi: 10.1186/s12964-025-02160-9 (PMC11967126; doi:10.1186/s12964-025-02160-9)
Supplement: Supplementary file 1 — Supplementary Material 1 [file 12964_2025_2160_MOESM1_ESM.docx]

**Atovaquone-Induced Activation of the PERK/eIF2α Signaling Axis Mitigates Metabolic Radiosensitisation**

*Jie Feng* *^a^, Varun Pathak ^b^, Niall M Byrne ^a^, Sarah Chambers, Tongchuan Wang ^a^, Rayhanul Islam ^a^, Reinhold J. Medina ^b,c^ , Jonathan A Coulter*^, a,^*

a. School of Pharmacy, Queens University Belfast, Queen’s University Belfast, Northern Ireland, UK

b. Welcome-Wolfson Institute for Experimental medicine, Queen’s University Belfast, Northern Ireland, UK

c. Institute for Life Course and Medical Sciences, Department of Eye and Vision Sciences, University of Liverpool, Liverpool, UK

*Corresponding author:

Jonathan Coulter, School of Pharmacy, Queens University Belfast, Lisburn Road, Belfast BT9 7BL, UK. Email: j.coulter@qub.ac.uk

**Supplementary Methods**

**Direct toxicity of Atovaquone assessed by Alamar blue assay**

The direct toxicity of atovaquone was assessed using Alamar Blue reagent. Briefly, 1x10^4^ cells were seeded in each well of a 96-well plate. After 24 h, cells were incubated with atovaquone at concentrations ranging from 2.5 to 150 μM. After a 20 h treatment period cells were washed with PBS and incubated with 10% Alamar Blue for 4 h, after which 100 μL of cell culture media was transferred to a black plate. Fluorescence levels were read at 570 nm excitation and 585 nm emission using the flurostar plate reader. Cell viability was assessed by comparing the fluorescence values of each treatment group to that of the vehicle only control group.

**Quantitative reverse transcription-PCR**

Quantitative PCR data was generated using a Roche LightCycler and SYBER Green I Mastermix (Roche, SUI) using the following setting: activation, hold 95°C for 15 min; amplification (95°C for 30 s; 58°C for 10 s; 72°C for 10 s. Fluorescence measurements were collected over 50 cycles, with primer specificity confirmed by the presence of a single melt curve peak. All values were normalised to the housekeeper RPL13 using the 2^−△CT^ method. Primer sequence: HIF-1α For-5’-ATCACCCTCTTCGTCGCT-3’, HIF-1α Rev-5’-GGAAAGGCACCAGAGGT-3’, RPL13A For-5’-TGGTCGTACGCTGTGAAGG-3’, RPL13A Rev- 5’-AGGAAAGCCAGGTACTTCAACTT-3’, PDK1 For-5’-AGTTCATGTCACGCTGGGTA-3’, PDK1 Rev-5’-CAGCTTCAGGTCTCCTTGGA-3’, CAIX For-5’-AGTTGCTGTCTCGCTTGGAA-3’, CAIX Rev-5’-TCGGAAGTTCAGCTGTAGCC-3’.


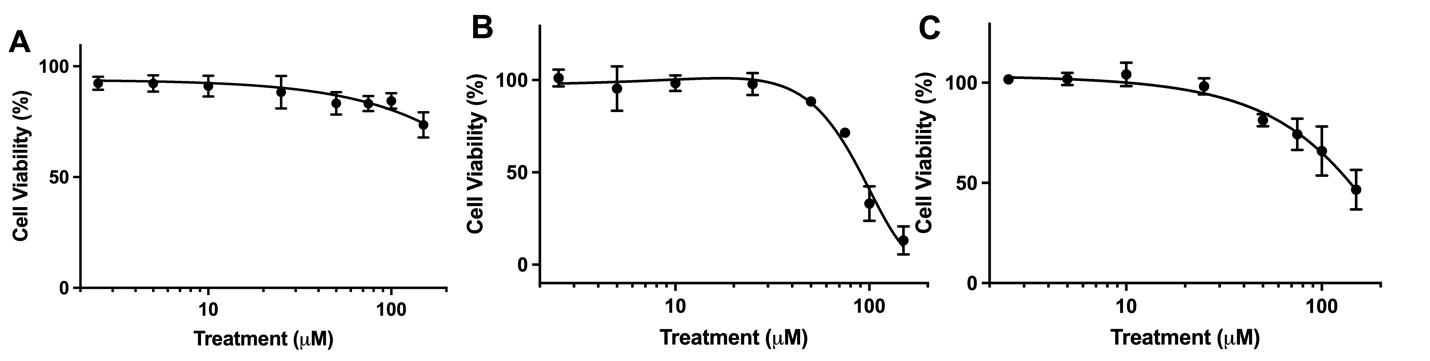
**Supplementary Data**

**Figure S1. Direct toxicity of atovaquone in HNSCC cells.** **A)** FaDu, **B)** CAL27 and **C)** CAL33 cells were treated with atovaquone at increasing concentrations for 20 h. Cytotoxicity was determined using an Alamar blue assay.

**
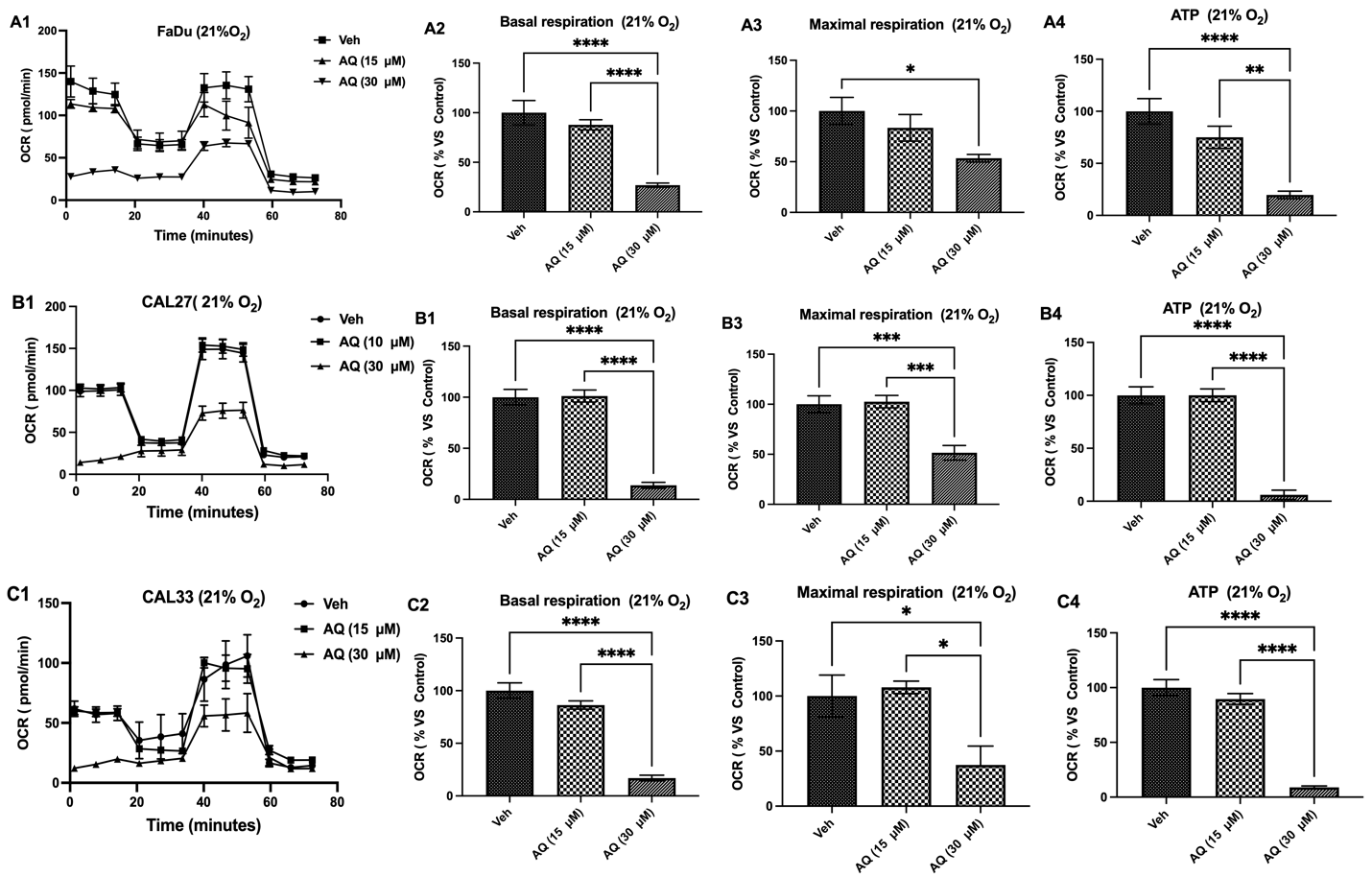
**

**Figure S2. Atovaquone treatment inhibited mitochondrial respiration of HNSCC cell under normoxia.** The metabolic profile of normoxic: **A1)** FaDu, **B1)** CAL27 and **C1)** CAL33 cells treated with atovaquone (15 μM or 30 μM) for 5 h was analysed using the Seahorse XFe96 Analyser. Significant reductions in **A-C2)** basal respiration, **A-C3)** maximal respiration and **A-C4)** ATP levels were observed. Data represents mean +/- SEM of n=3 independent experiments. Statistical differences were determined using one-way ANOVA with a Tukey multiple comparison test, with ** representing p<0.01 and **** p<0.0001.


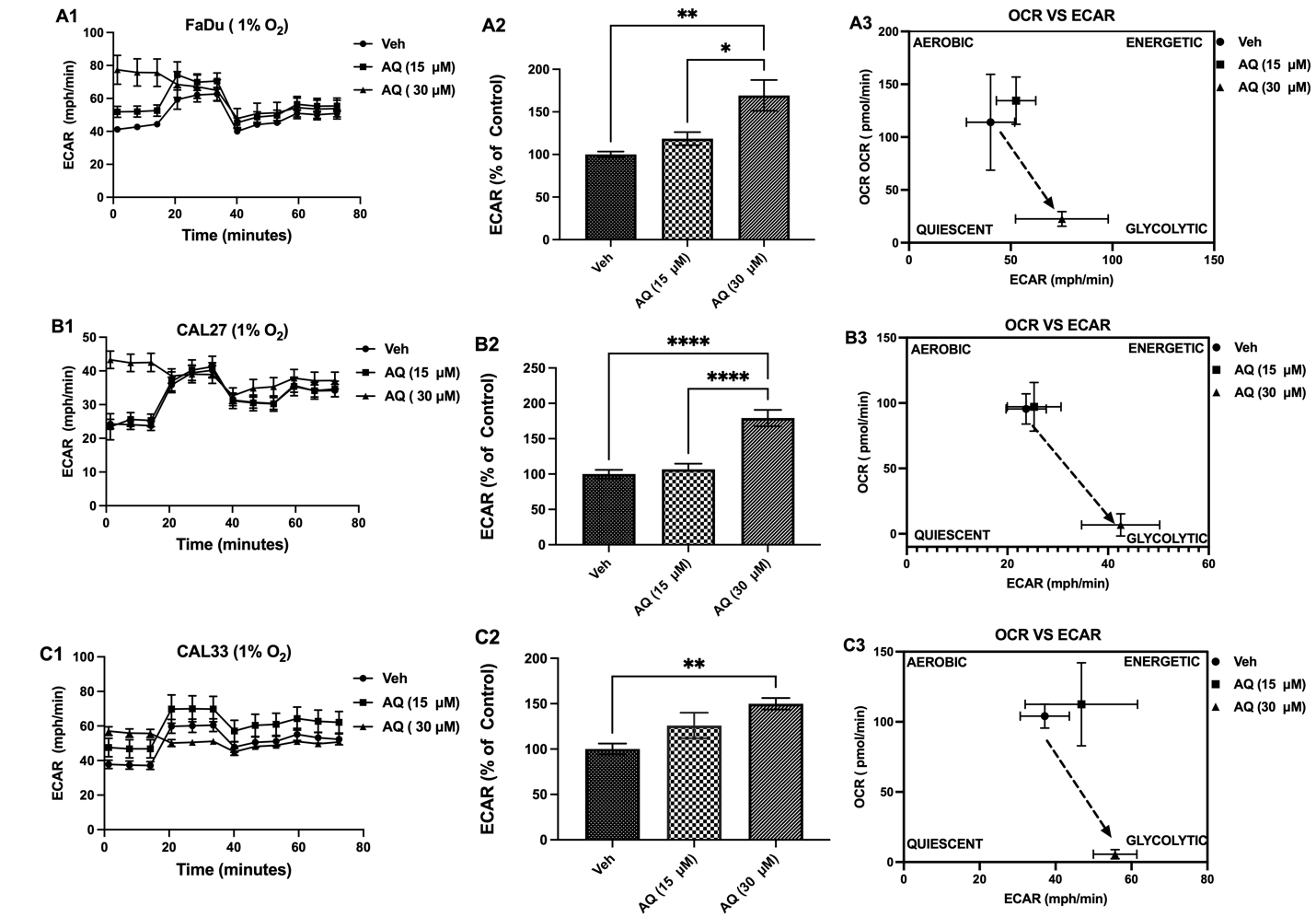


**Figure S3 Atovaquone treatment induced glycolysis in HNSCC cells under hypoxia (1% O_2_).** **A1-C1).** Seahorse analysis of extracellular acidification rate (ECAR) in FaDu, CAL27 and CAL33 cells after hypoxic acclimatisation (1 h pre-exposure to 1% O_2_) and a 5 h atovaquone treatment under hypoxia prior to analysing by the Seahorse XFe96 Analyser. **A2-C2)** Graphical representation of basal ECAR levels following atovaquone treatment. **A3-B3)** Phenograms representing the atovaquone induced switch from aerobic to glycolytic respiration, generated by plotting OCR against ECAR. Statistical differences were determined using one-way ANOVA with a Tukey multiple comparison test, with **p* < 0.01, ***p* < 0.001, *****p* < 0.00001.


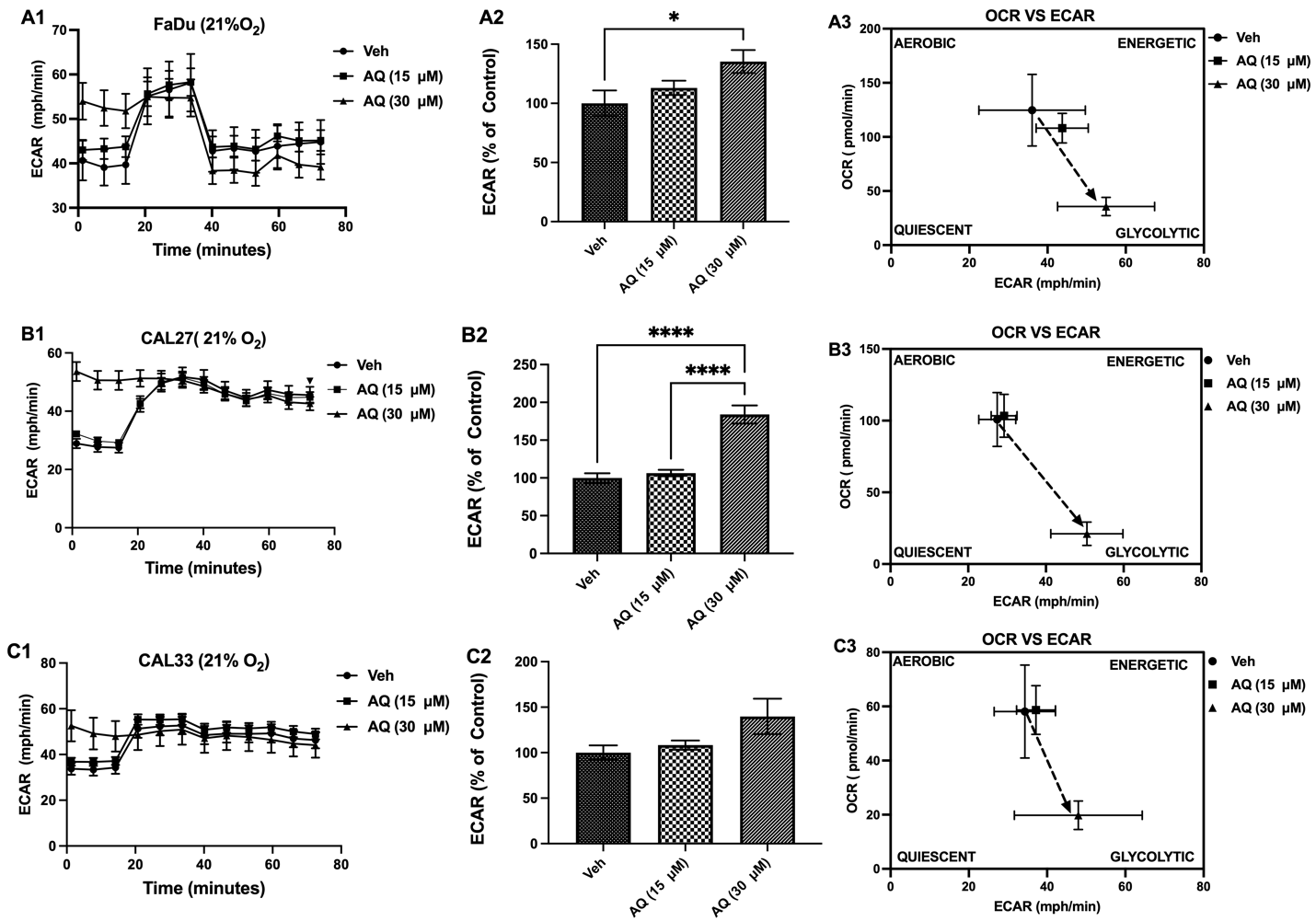


**Figure S4 Atovaquone treatment induce glycolysis in HNSCC cells under normoxia (21% O_2_).**  **A1-C1).** Seahorse analysis of extracellular acidification rate (ECAR) in FaDu, CAL27 and CAL33 cells under normoxic conditions (21% O_2_) following a 5 h atovaquone treatment. **A2-C2)** Graphical representation of elevated basal ECAR following atovaquone treatment. **A3-B3)** Phenograms representing the atovaquone induced switch from aerobic to glycolytic respiration, generated by plotting OCR against ECAR. Statistical differences were determined using one-way ANOVA with a Tukey multiple comparison test, with **p* < 0.01, *****p* < 0.00001.


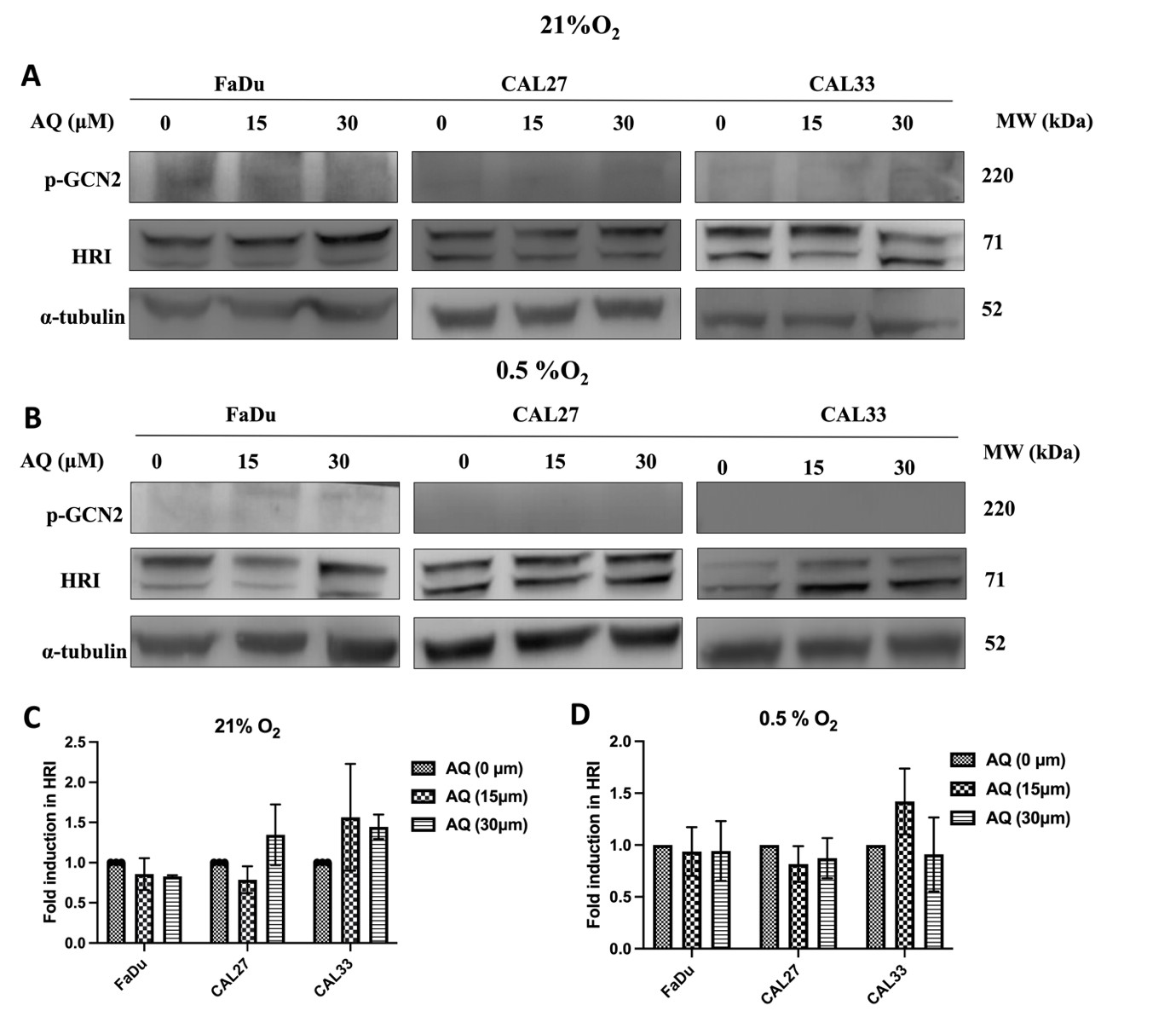
**Figure S5. Effect of Atovaquone on ISR signaling under normoxia and hypoxia.** FaDu, CAL27 and CAL33 cells were treated with atovaquone (30 μM) for 5 h under **A)** atmospheric oxygen (21% O_2_) or **B)** hypoxia (0.5% O_2_), then analysed by western blot for alterations in p-GCN2, HRI and α-tubulin, using the latter as the loading control. Quantitative densitometric analysis for: **C)** normoxic (21% O_2_) p-GCN2 and HRI protein expression, and **D)** hypoxic (0.5% O_2_) p-GCN2 and HRI protein expression. Blots and quantified data are representative of the mean of a minimum of three independent experiments ± SEM. Statistical differences were determined using one-way ANOVA with a Tukey multiple comparison test, with * representing p ≤ 0.05, *** p ≤ 0.001.

**
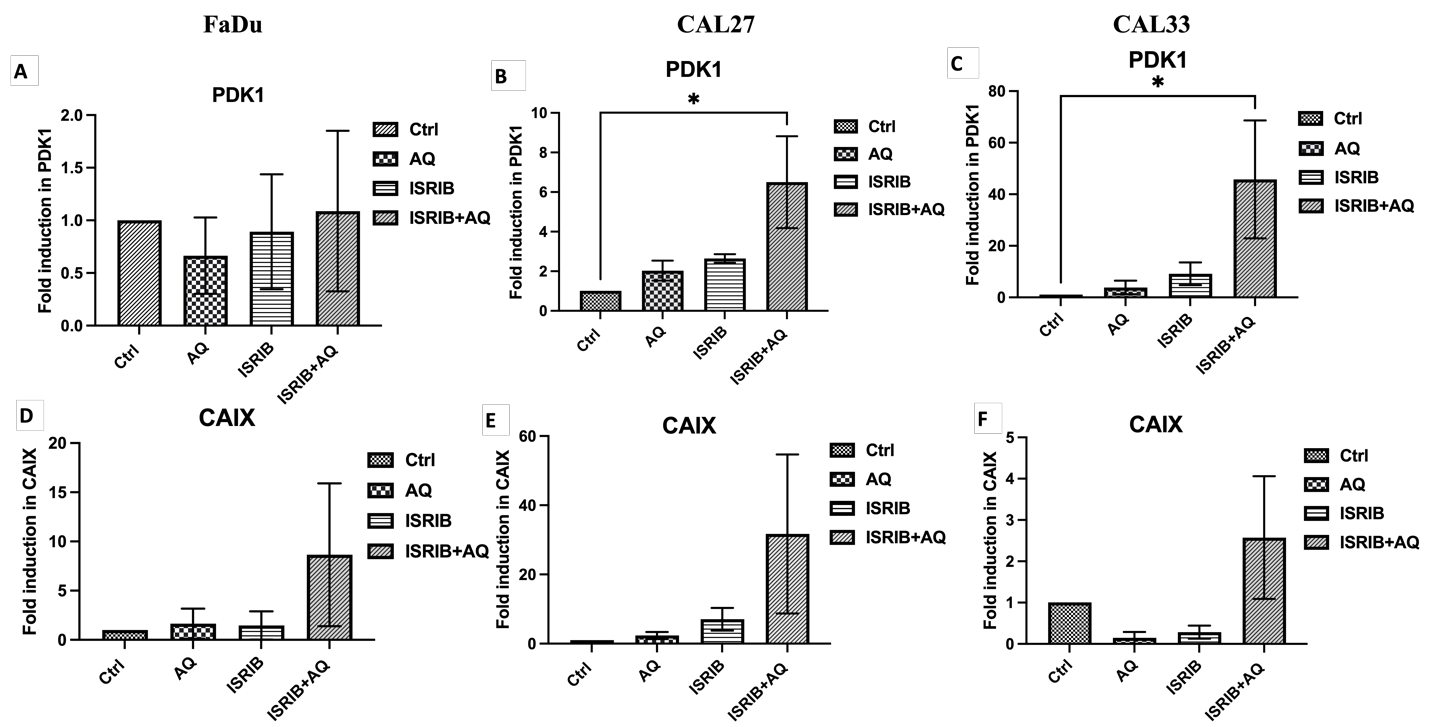
**

**Figure S6. Atovaquone activation of ISR suppresses PDK1 and CAIX, key HIF-target genes.** Quantitative RT–PCR analysis of **A-C)** PDK-1 and **D-F)** CAIX mRNA determined using whole RNA isolates from hypoxic (0.5% O_2_ for 5 h) FaDu, CAL27 and CAL33 cells treated with atovaquone (30 μM), ISRIB (1 μM), or the combination of atovaquone and ISRIB. All vaule are normalised against housekeeper RPL13A mRNA with fold changes calculated against untreated hypoxic controls. Data are representative of the mean of three independent experiments ± SEM. Statistical differences were determined using one-way ANOVA with a Tukey multiple comparison test, with * representing p ≤ 0.05.

**
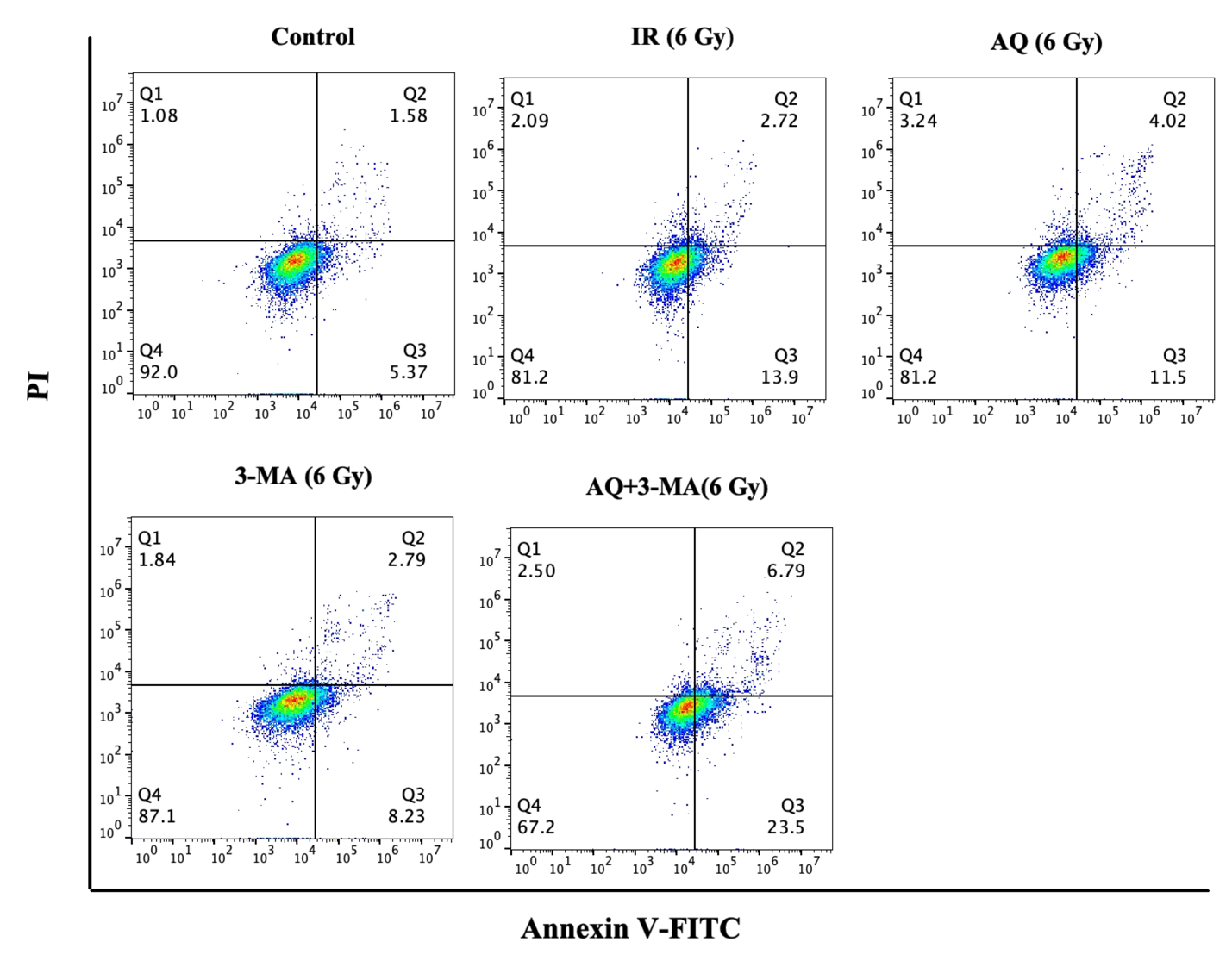
**

**Figure S7. Flow cytometry analysis of apoptosis induction following atovaquone treatment and suppressed autophagy.** Representative dot plot data of Annexin-V/PI flow cytometry analysis of CAL27 cells following treatment with atovaquone (AQ 30 μM) and/or 3-MA (2 mM) alone or in combination with radiation (6 Gy) under hypoxia (0.5% O_2_ for 5 h). Experiments were performed in triplicate and data is expressed as mean ± SEM.

**Supplementary Table 1.** Oxygen Enhancement Ratio (OER) of HNSCC cells after treatment with atovaquone under hypoxia (0.5% O_2_). OER is defined as a ratio of the radiation doses required under two differing oxygen tensions to achieve the same biological effect. In the current work this was defined as normoxia (21% O_2_ verses hypoxia 0.5% O_2_) reducing surviving fraction (SF) to 0.5 and 0.1, i.e. 50% and 90% cell kill. OER data was extrapolated from the clonogenic survival data presented in Figure 2.
