# Supplementary material for: Atovaquone-induced activation of the PERK/eIF2α signaling axis mitigates metabolic radiosensitisation
Source: Cell Commun Signal. 2025 Apr 2;23:164. doi: 10.1186/s12964-025-02160-9 (PMC11967126; doi:10.1186/s12964-025-02160-9)

Western Blot images for Figure 4

Figure 4a. Replicates 1-3

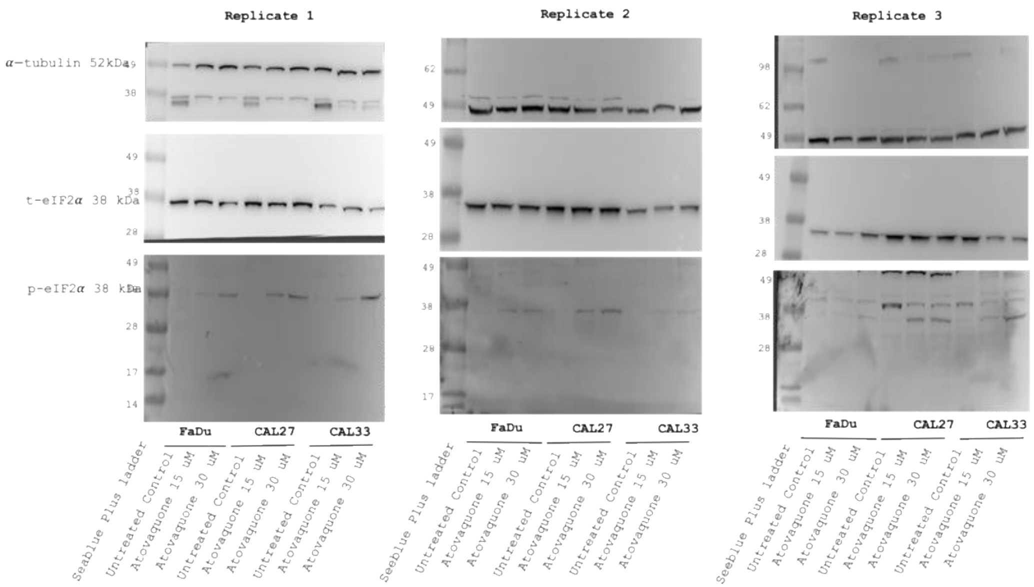

Figure 4b. Reps 1-3/4

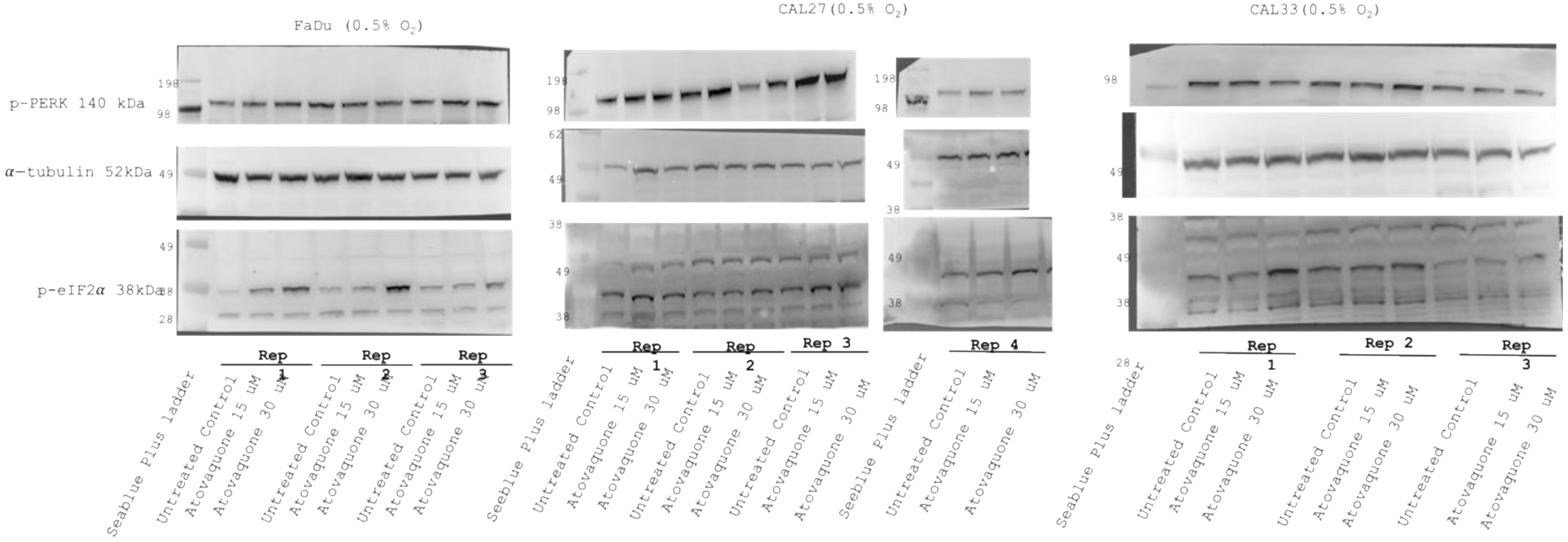

Western Blot images for Figure 5

Figure 5 G – Reps 1-3

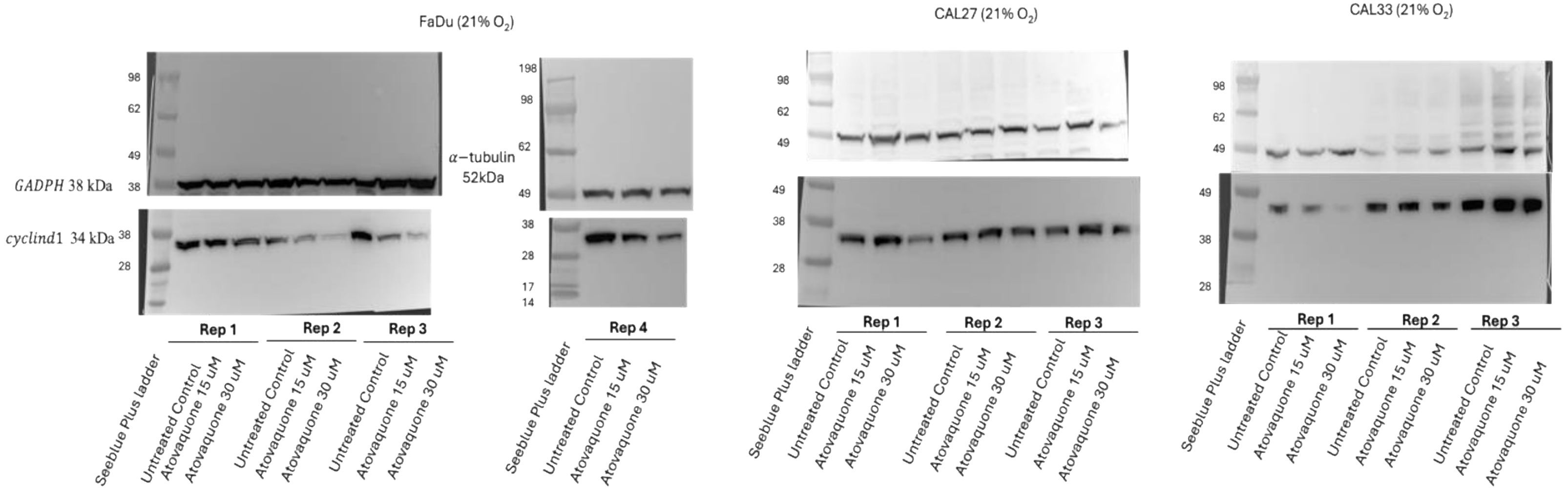

Figure 5 H – Reps 1-3

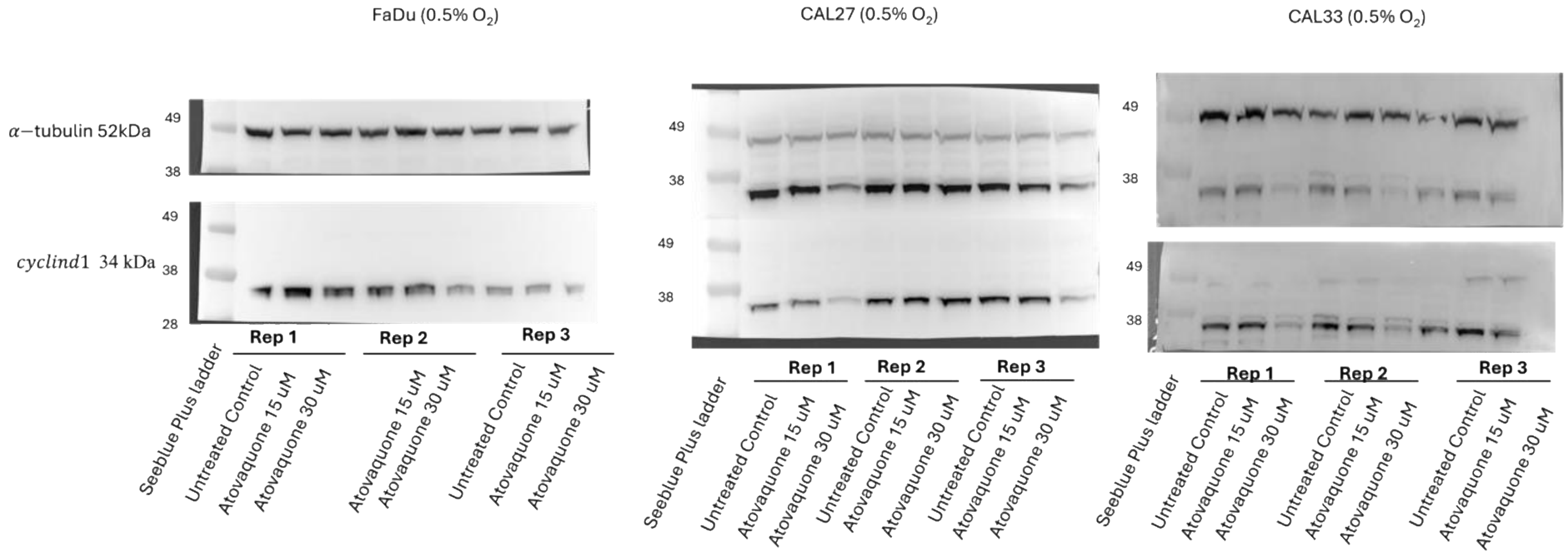

Western Blot images for Figure 6

Figure 6a. Replicates 1-3

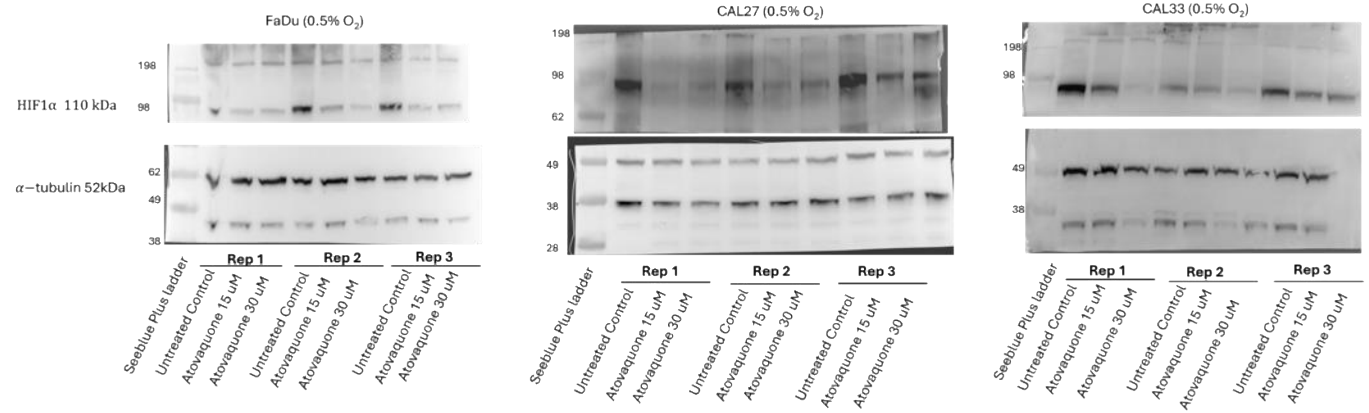

Figure 6c. Replicates 1-3

HIF1a

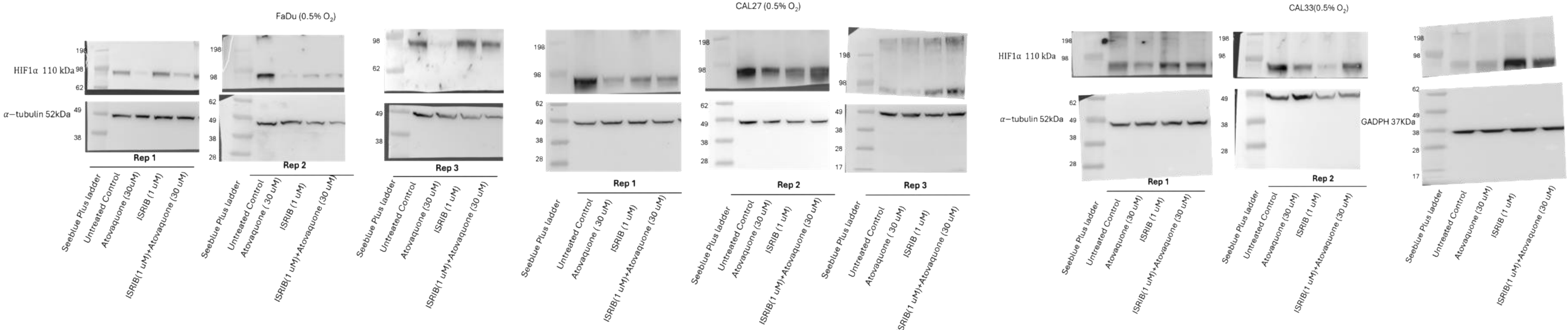

eIF2a

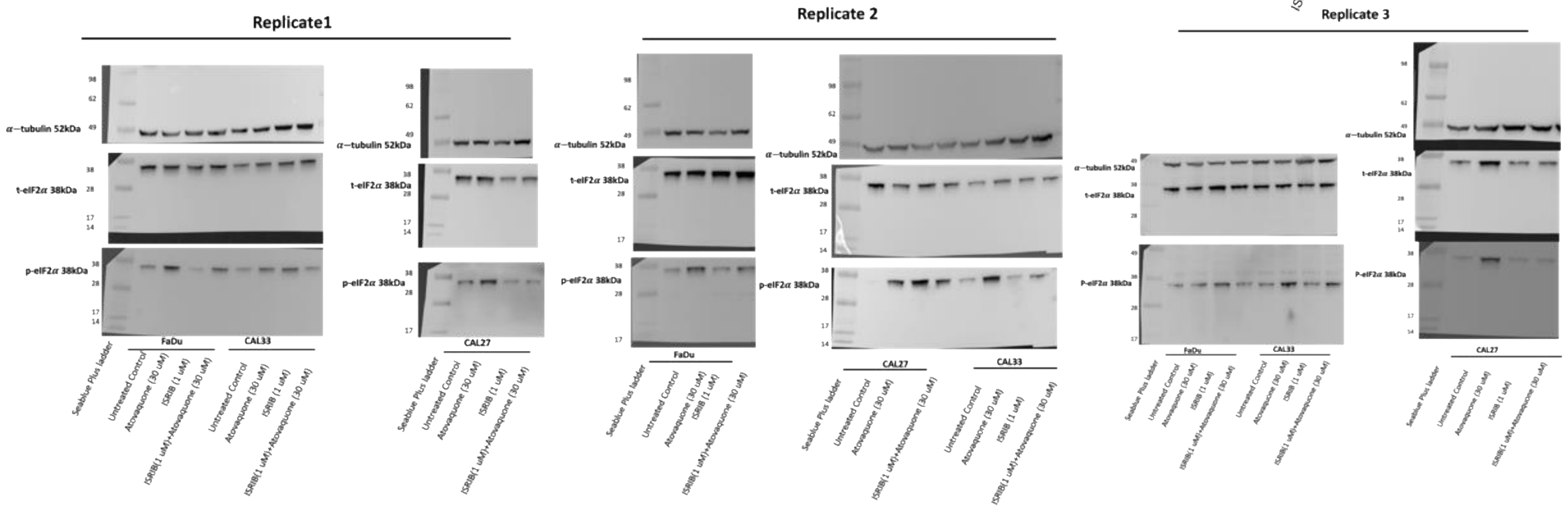

Western Blot images for Figure 7

Figure 7a. Replicates 1-3

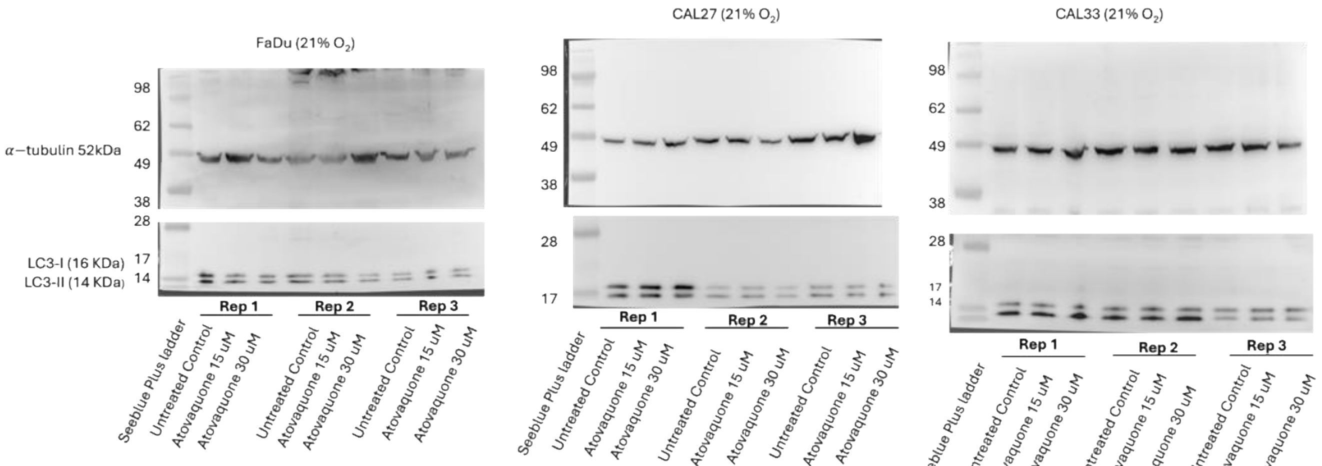

Figure 7b. Replicates 1-4
(notes: replicate 1 all three cell lines on one membrane; CAL27 Replicates 2-4 on separate membranes; CAL33 Replicates 2-4 on one membrane.

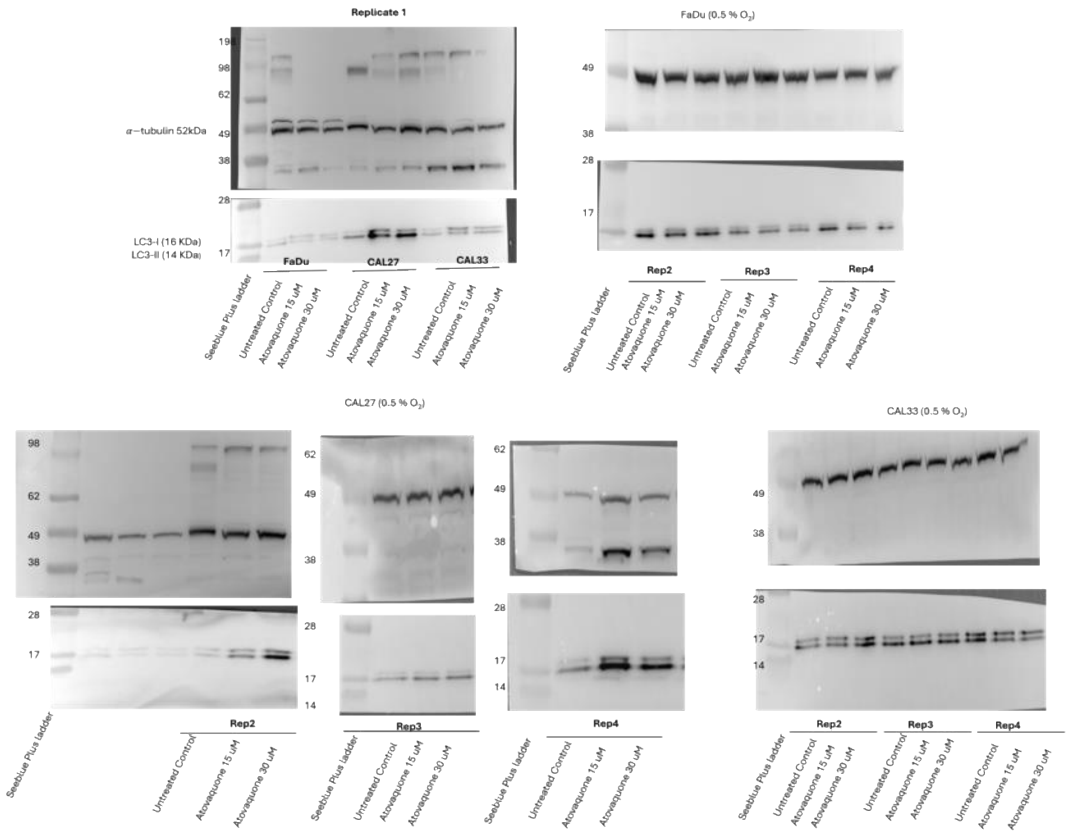

Western Blot images for Figure 8

Figure 8a. Replicates 1-3

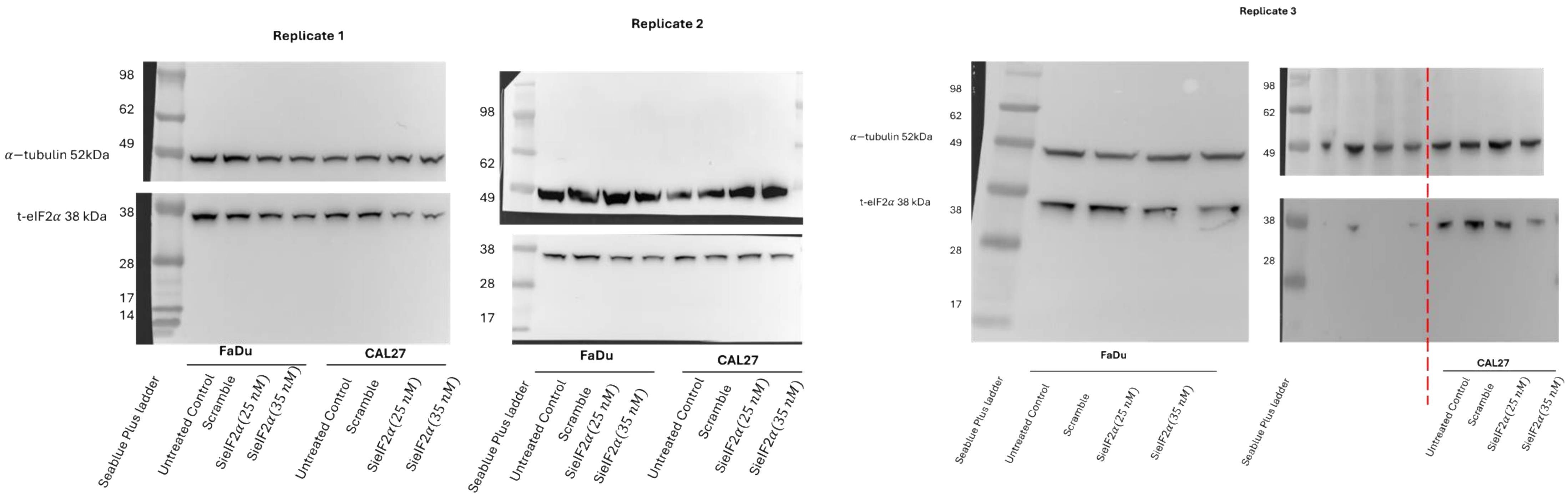

Western Blot images for Supplementary Figure S3

Figure S3. Replicates 1-3

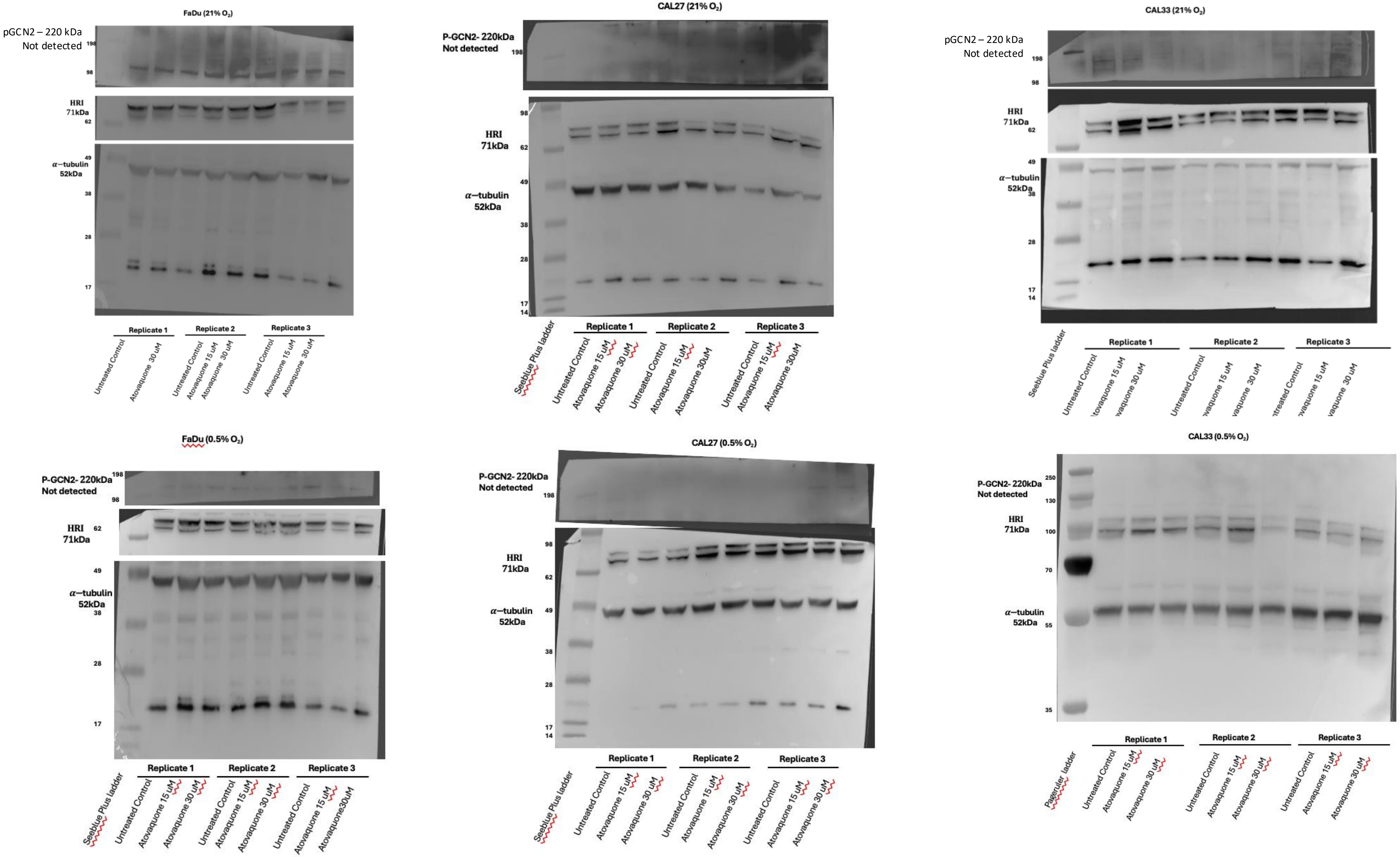

Supplement: Supplementary file 2 — Supplementary Material 2 [file 12964_2025_2160_MOESM2_ESM.pdf]
